# Breakfast in Japan: Findings from the 2012 National Health and Nutrition Survey

**DOI:** 10.3390/nu10101551

**Published:** 2018-10-19

**Authors:** Kentaro Murakami, M. Barbara E. Livingstone, Aya Fujiwara, Satoshi Sasaki

**Affiliations:** 1 Department of Social and Preventive Epidemiology, School of Public Health, University of Tokyo, Tokyo 113-0033, Japan; stssasak@m.u-tokyo.ac.jp; 2 Nutrition Innovation Centre for Food and Health (NICHE), School of Biomedical Sciences, Ulster University, Coleraine BT52 1SA, UK; mbe.livingstone@ulster.ac.uk; 3 Department of Social and Preventive Epidemiology, Graduate School of Medicine, University of Tokyo, Tokyo 113-0033, Japan; fujiwaraay-tky@umin.ac.jp

**Keywords:** breakfast, diet quality, nutrient intake, food intake, NRF9.3, Japan

## Abstract

We assessed breakfast in Japan using data from the 2012 National Health and Nutrition Survey. Dietary data were obtained from 1444 children (aged 6–11 years), 1134 adolescents (aged 12–17 years), 6531 younger adults (aged 18–49 years), and 13,343 older adults (aged ≥ 50 years), using a one-day weighed dietary record. Overall, 97% of participants reported consuming breakfast. Compared with breakfast skippers, breakfast consumers had a higher daily diet quality score assessed by the Nutrient-Rich Food Index 9.3 (NRF9.3). For those who consumed breakfast, breakfast accounted for 20–25% of daily energy intake. In comparison with the contribution to energy, breakfast accounted for higher proportions of carbohydrate and riboflavin, and lower proportions of MUFA, *n*-3 PUFA, thiamin, and niacin, as well as vitamins B-6 and C. The overall diet quality (NRF9.3 score) was positively associated with breakfast intake of protein, *n*-6 PUFA, *n*-3 PUFA, carbohydrate, dietary fiber, and almost all micronutrients examined, and inversely with that of added sugar. For foods, the NRF9.3 score was positively associated with breakfast intake of rice, potatoes, pulses, vegetables, fruits, and eggs and inversely with that of bread, sugar, and soft drinks. The findings will be useful in developing dietary recommendations for a balanced breakfast among Japanese.

## 1. Introduction

Breakfast is often considered the most important meal of the day, and an important part of a healthy lifestyle for both children and adults. Much evidence has demonstrated that the consumption of breakfast is associated with higher quality diets in terms of key nutrients and favorable food groups [1,2,3,4,5,6,7,8]. In comparison, skipping breakfast is associated with a lower quality diet and unfavorable metabolic risk factor profiles [9,10,11,12,13,14,15,16,17].

To our knowledge, research on breakfast consumption patterns based on detailed dietary intake information has not been conducted in Japan. Generational differences in breakfast consumption are also largely unknown. The Japanese diet has long been of interest in other countries, partly owing to the lower prevalence of coronary artery disease and longer life expectancy of Japanese [18,19]. Recently, typical Japanese diets have come to include a high intake of refined grains (primarily white rice), seaweeds, vegetables, fish, soy foods, and green tea, and a low intake of whole grains, processed meat, nuts, and soft drinks [20,21]. The typical Japanese meal pattern consists of three meals (breakfast, lunch, and dinner), as well as snacks. Breakfast is typically based on rice or bread as a staple food, accompanied by other foods such as vegetables, fruits, dairy products, pulses, eggs, and tea and coffee [22], which is somewhat different from that typically consumed in Western countries [1,2,3,4,5,6,7].

More recently still, the establishment of the International Breakfast Research Initiative (IBRI), a trans-Atlantic international collaborative study, represents a notable step in investigating the nutritional impact of breakfast. Using a harmonized analytical approach, the IBRI aims to develop evidence-based recommendations, primarily aimed at ensuring a nutrient-based balanced breakfast [1]. Results from national representative samples in Canada [2], the UK [3], France [4], Denmark [5], the US [6], and Spain [7] have been published. Adoption of this harmonized approach by other countries such as Japan will facilitate more informed and consistent cross-country evaluation of the nutritional characteristics of breakfast than has hitherto been the case.

Accordingly, we conducted the present cross-sectional study by following the analytic plan used in IBRI project [1]. Based on one-day dietary data from the National Health and Nutrition Survey, Japan (NHNSJ), our purpose was: (1) to assess the prevalence of breakfast skipping; (2) to compare daily intakes of energy, nutrients, and food groups and overall diet quality between breakfast skippers and consumers; (3) to assess intakes of energy, nutrients, and food groups at breakfast as well as the contribution of breakfast to daily energy and nutrient intake among breakfast consumers; and (4) to examine the association between overall diet quality and intakes of energy, nutrients, and food groups at breakfast. In this study, we hypothesized that overall diet quality is higher in breakfast consumers compared with breakfast skippers, and that, among breakfast consumers, higher overall diet quality is associated with better breakfast intake. We anticipated that the study would serve as the first scientific basis for the development of dietary recommendations for a balanced breakfast for Japanese.

## 2. Materials and Methods

### 2.1. Data Source and Analytic Sample

The NHNSJ is a national nutrition survey conducted annually since 1945 by public health centers with the supervision of the Ministry of Health, Labour and Welfare in accordance with the Health Promotion Law. Our present cross-sectional analysis used data from the 2012 NHNSJ, with permission (because the 2012 survey is larger in sample size than more recent and available one). The NHNSJ have been detailed elsewhere [21,23]. Briefly, based on the population census, 475 of the approximately one million census units were sampled randomly as survey areas. All non-institutionalized Japanese aged 1 year or older living in survey areas (approximately *n* = 61,000) were asked to participate, excluding individuals holding foreign citizenship, those whose diet was not self-selected, and those using a special diet (mainly due to disease). A total of 12,750 of 24,555 eligible households (52%) participated. The survey was conducted between 25 October and 7 December 2012.

The number of individual participants in the dietary survey part of NHNSJ 2012 was 32,228. Consistent with the IBRI project [2,3,4,5,6,7], we excluded data from individuals aged ≤ 5 years (*n* = 1370); lactating or pregnant women (*n* = 368), because they were presumed to be not following their usual diet; and individuals with missing information on the variables of interest (*n* = 8038, mainly body height or weight). The final analysis sample consisted of 22,452 individuals, who were divided into four age groups for analysis: children (aged 6–11 years; *n* = 1444), adolescents (aged 12–17 years; *n* = 1134), younger adults (aged 18–49 years; *n* = 6531), and older adults (aged ≥ 50 years; *n* = 13,343).

The NHNSJ was conducted in accordance with the guidelines of the Declaration of Helsinki. Verbal informed consent was obtained from all participants. Under the Statistics Act, the Ministry of Health, Labour and Welfare anonymized individual-level data collected from the NHNSJ and provided the first author with the datasets for this study. Under the requirements of the Ethical Guidelines of Epidemiological Research by the Ministry of Education, Culture, Sports, Science and Technology and the Ministry of Health, Labour and Welfare, institutional review board approval for this analysis was not required.

### 2.2. Dietary Assessment

Dietary intake data were acquired using one-day weighed household dietary records, as described previously [24,25,26,27], and therefore included data on all members of the household. For each household, a trained fieldworker (registered dietitian) supplied the main record-keeper with a diary for recording and received written and verbal instructions in the home on how the diary should be filled in. The main record-keeper was requested to weigh and record food and beverage items (except drinking water) consumed by each household member on the recording day. Equipment to weigh foods and beverages is usually already present in Japanese households, and was not provided, primarily due to limited funding. Most commonly, the main record-keeper was requested to weigh all ingredients used in food preparation. In cases where household members shared food items from a single dish, the record-keeper was asked to record approximate proportions of food eaten by individual members so that the dietary intake of each individual could be estimated. If weighing was not possible (e.g., eating out), the record-keeper was asked to record intakes in as much detail as possible, including estimated portion size, as well as any leftovers. To maximize involvement, the household was permitted to select the recording day freely, excluding Sundays, national holidays, and days with special events (weddings, etc.). Although we did not collect identity information of the main record-keeper, we assumed in the survey that recording was done by the main household cook, who in Japan is most commonly a woman. Very shortly after recording (usually the next weekday), each household was visited by trained fieldworkers, who collected the diary, confirmed the completeness of food recording, and obtained any additional information as necessary.

Following the NHNSJ study manual, trained fieldworkers changed estimated portion sizes into weights (for food items recorded using household measures), and added a code for all individual food items based on the Standard Tables of Food Composition in Japan [28]. The collected dietary records were then reviewed at the local center, and the data were input by trained fieldworkers using software specially developed for the NHNSJ. The data were then compiled by trained investigators at the central office to prepare the overall dietary dataset. Estimated daily intakes of foods, energy, and nutrients for individuals were calculated using the household food consumption record, and for shared dishes and foods, approximate proportions consumed by each household member, based on the Standard Tables of Food Composition in Japan [28]. Added sugar was estimated based on a recent comprehensive composition database [29,30]. Dietary supplements were not considered during the nutrient intake calculation because it was our intention to assess only nutrient intake from foods and beverages.

The validity of this household dietary record in estimating individual-level dietary intake among Japanese has been investigated [27]. Dietary intake by young women (aged about 20 years) estimated using this one-day household dietary record conducted by their mother (mean age: 49 years) was compared against intake estimated using a one-day weighed dietary record, which was independently conducted by the young woman herself (*n* = 32). Differences in mean intake between these methods were 6.2% for energy; 5.7% for protein; 6.7% for fat; and 6.3% for carbohydrate. In addition, Pearson correlation coefficients were 0.90 for energy, 0.89 for protein, 0.91 for fat, and 0.90 for carbohydrate. Moreover, mean values for the ratio of energy intake to estimated energy requirement in previous analyses using the NHNSJ 2012 were 1.04 for children [31] and 0.98 for adults [32].

### 2.3. Assessment of Overall Diet Quality

Consistent with the IBRI project [1], overall diet quality was measured using the Nutrient-Rich Food Index 9.3 (NRF9.3) score, as described in detail elsewhere [33,34,35,36]. In short, the NRF9.3 is a validated, composite measure of the nutrient density of the total diet, calculated as the sum of the percentage of reference daily values (DVs) for nine qualifying nutrients (protein, dietary fiber, vitamins A, C, and D, calcium, iron, potassium, and magnesium) minus the sum of the percentage of DVs for three adverse nutrients (added sugar, saturated fat, and sodium). Considering the differences in dietary intake and habits between Japan and Western countries, DVs were determined (for sex and age categories) based on the Dietary Reference Intakes (DRIs) for Japanese, 2015 [37] (as shown in Table S1), namely the Recommended Dietary Allowance for protein, vitamins A and C, calcium, iron, and magnesium; and tentative dietary goal for preventing lifestyle-related diseases for dietary fiber, potassium, saturated fat, and sodium. In terms of added sugar, the conditional recommendation advocated by the World Health Organization (i.e., an upper limit of 5% of energy) [38] was used because of the lack of a recommended value for added sugar in Japan as well as its low intake [29,30]. The overall daily intake of each nutrient for each participant was normalized for the sex- and age-specific Estimated Energy Requirement for a moderate level of physical activity (from DRIs) and expressed as a percentage of the DV. For qualifying nutrients, each percentage of DVs was terminated at 100 such that a high intake of one nutrient would not compensate for the low intake of another. With regard to adverse nutrients, consideration was limited to the share which exceeded the recommended amount. Accordingly, higher NRF9.3 scores indicate a better quality of the overall diet, and a maximum possible score of 900 indicated a diet in which intakes per given amount of energy were above the DVs for the nine qualifying nutrients but were below the DVs for the three disqualifying nutrients. As expected, after adjustment for age, sex, weight status, and occupation (associations with NRF9.3 score are shown in Table S2), as well as total energy intake, a higher NRF9.3 score was associated with favorable patterns of overall diet in all four populations (Table S3 for macronutrients, Table S4 for vitamins and minerals, and Table S5 for food groups), suggesting the usefulness of the NRF9.3 score as a measure of overall diet quality in Japanese.

### 2.4. Definition of Breakfast Consumption

The NHNSJ food diary sheet reflected a typical Japanese eating pattern, consisting of breakfast, lunch, and dinner, as well as snacks. Breakfast intake in the present study was therefore based on self-reporting, and included the total of foods and beverages recorded in the breakfast section. In addition, individuals who reported that they did not consume any foods or beverages at breakfast on the recording day were categorized as breakfast skippers, while those who did report consumption of any food or beverage at breakfast on the recording day were considered breakfast consumers. For consumers, intakes of food groups, nutrients, and energy at breakfast were calculated, with the same procedure used to calculate the overall daily dietary intake (See Section 2.2).

### 2.5. Assessment of Basic Characteristics

In the present study, anthropometric measurements were performed for 69% of the participants by trained fieldworkers using standardized procedures. Body height (nearest 0.1 cm) and weight (nearest 0.1 kg) were measured while the participants were barefoot and wearing light clothes only. Of the remaining 31% of participants, two variables were measured by other household members at home or were self-reported. Body mass index (BMI; kg/m^2^) was calculated by the commonly used formula, namely weight (kg) divided by height squared (m^2^). The weight status of children and adolescents was defined using to the International Obesity Task Force for age- and sex-specific BMI cutoffs [39,40], corresponding to adult BMI < 17 for underweight, ≥17 to <25 for normal weight, and ≥25 for overweight/obese. Weight status in adults was defined based on BMI according to WHO recommendations [41], namely underweight < 18.5, normal weight ≥ 18.5 to < 25, and overweight/obese ≥25. Information on occupation (professional/manager, sales/service/clerical, security/transportation/labor, or not in paid employment) was obtained from adult participants using a self-administered questionnaire.

### 2.6. Statistical Analysis

All statistical analyses for each of the four populations were performed using SAS statistical software (version 9.4, SAS Institute Inc., Cary, NC, USA). Data are presented as means ± standard deviation (or standard deviation for adjusted models) for continuous variables and as the numbers and percentages of participants for categorical variables. Differences in basic characteristics between breakfast skippers and consumers were examined based on the independent *t*-test (for age) and chi-square test (for sex, weight status, and occupation). Differences in daily dietary intakes (energy, nutrients, and food groups) between breakfast skippers and consumers were examined using general linear models with adjustment for these basic characteristics as well as total energy intake (except for the analysis on energy itself). Thus, we constructed multivariate nutrient density models for macronutrients expressed as percent of energy and standard multivariate models for all other dietary variables [42]. Food grouping was based on the similarity of nutrient profiles or culinary usage of the foods, mainly according to the Standard Tables of Food Composition in Japan [38] and the classification of food groups used in the NHNSJ [21]. The nutrients examined (See Tables S3 and S4) were those presented in the Japanese DRIs [37] (except for five nutrients without sufficient food composition tables in Japan: biotin, iodine, selenium, chromium, and molybdenum) as well as added sugar. After limiting analysis to data on breakfast consumers, dietary intake at breakfast as well as the contribution of breakfast to daily energy and nutrient intakes were described. Finally, breakfast intake was examined according to tertile category of NRF9.3 score as a measure of overall diet quality, using general linear models with adjustment for age, sex, weight status, and occupation as well as total energy intake (except for the analysis on energy itself). All reported *p* values are two-tailed and *p* values < 0.05 were considered statistically significant.

## 3. Results

### 3.1. Prevalence of Breakfast Skipping and Consumption

In the present study based on the 2012 NHNSJ (1444 children, 1134 adolescents, 6531 younger adults, and 13,343 older adults), the majority of participants reported consuming breakfast on the diet recording day (99.3%, 97.4%, 92.3%, and 98.4%, respectively; overall 96.7%) (Figure S1). Basic characteristics of breakfast skippers and consumers are shown in Table 1. Among adolescents, breakfast consumers were younger than breakfast skippers. Among younger and older adults, breakfast consumers were more likely to be older, be female, be in the range of normal weight (younger adults only), and not in paid employment.

### 3.2. Daily Intake of Breakfast Skippers and Consumers

For all four age groups, overall diet quality as assessed by the NRF9.3 score was higher in breakfast consumers than skippers after adjustment for potential confounding factors (age, sex, weight status, occupation, and energy intake) (Table S6). Breakfast consumers also had a higher daily energy intake in all age groups (except for children). In terms of macronutrients, there was no significant difference between breakfast consumers and skippers in children or adolescents, except for higher absolute intakes of protein and dietary fiber (adolescents only) and a higher percentage of energy from protein (children only) in breakfast consumers. In younger and older adults, breakfast consumers had higher absolute intakes of protein (younger adults only), carbohydrate, and dietary fiber and lower intakes of fat, SFA, MUFA, and added sugar, as well as a higher percentage of energy from carbohydrate and lower percentages from fat and MUFA.

Irrespective of age, overall diets of breakfast consumers were generally high in vitamins and minerals compared with those of breakfast skippers (Table S7). For example, breakfast consumers had higher intakes of vitamin K, folate, vitamin C, calcium, magnesium, and phosphorus in all age groups and higher intakes of vitamin B-6, pantothenic acid, potassium, and iron in three age groups. For food groups (Table S8), overall diets of breakfast consumers were lower in confectioneries among all four age groups and higher in bread, vegetables, fruits, eggs, and dairy products and lower in soft drinks in three age groups.

### 3.3. Breakfast Intake and Contribution of Breakfast to Daily Energy and Nutrient Intake

**Figure 1:**
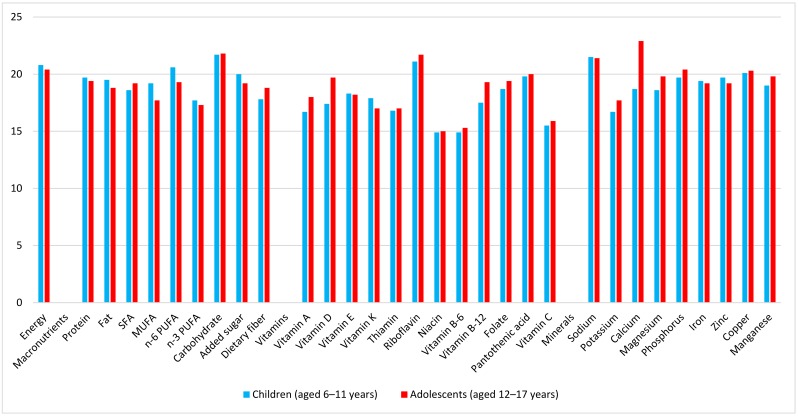
Percentage contribution of breakfast to daily intakes of energy, macronutrients, vitamins, and minerals in children (aged 6–11 years; *n* = 1434) and adolescents (aged 12–17 years; *n* = 1104). Values are means.

**Figure 2:**
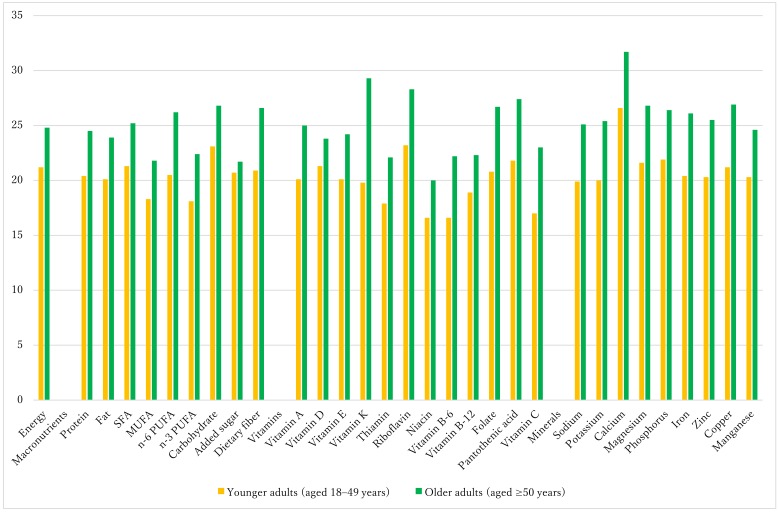
Percentage contribution of breakfast to daily intakes of energy, macronutrients, vitamins, and minerals in younger adults (aged 18–49 years; *n* = 6028) and older adults (aged ≥ 50 year; *n* = 13,135). Values are means.

The following analyses were limited to breakfast consumers. In all age groups, a variety of food groups were represented at breakfast, including rice, bread, sugar, pulses, vegetables, fruits, fish, meat, eggs, dairy products, fats and oils, tea and coffee, and seasonings (Table S9). Mean energy intake at breakfast was 374 kcal in children, 464 kcal in adolescents, 411 kcal in younger adults, and 472 kcal in older adults (Table S10), respectively, accounting for 20.8%, 20.4%, 21.2%, and 24.8% of total daily energy. Figure 1 shows the percentage contribution of breakfast to daily nutrient intakes in children and adolescents. Compared with the contribution of breakfast energy to the daily diet, breakfast contributed to a higher proportion of carbohydrate, riboflavin, sodium, and calcium (adolescents only). Conversely, the contribution of breakfast to SFA, MUFA, *n*-3 PUFA, dietary fiber, some vitamins (vitamins A, E, K, B-6, and C, thiamin, and niacin), and potassium was less than its contribution to energy. For younger and older adults (Figure 2), compared with its contribution to energy, breakfast contributed to a higher proportion of carbohydrate, riboflavin, and calcium but to a lower proportion of MUFA, *n*-3 PUFA, and several vitamins (thiamin, niacin, and vitamins B-6, B-12, and C). Additionally, in older adults, the contribution of breakfast to dietary fiber, vitamin K, folate, pantothenic acid, magnesium, and copper was higher than the contribution to energy, whereas its contribution to added sugar was lower.

### 3.4. Energy, Nutrient, and Food Group Intakes at Breakfast by Tertiles of Overall Diet Quality

To investigate the associations between breakfast intake and overall diet quality, participants were divided into tertiles according to the NRF9.3 score for each age group. After adjustment for potential confounding factors, the NRF9.3 score was positively associated with energy intake at breakfast in adolescents, and in younger and older adults, and also with the proportion of breakfast to total energy in all age groups (Table 2). In terms of macronutrients, the NRF9.3 score was associated positively with breakfast intake of protein, *n*-6 PUFA, *n*-3 PUFA, carbohydrate, and dietary fiber and inversely with that of added sugar, irrespective of age. Additionally, the proportion of protein to breakfast energy was higher while that of added sugar was lower among higher tertile categories of NRF9.3 score in all age groups.

Table 3 shows intakes of micronutrients at breakfast according to tertile category of the NRF9.3 score. There were positive associations between the NRF9.3 score and all vitamins and minerals examined in all four age groups (except for no associations for vitamin B-12 and sodium in children or adolescents, vitamin D in children, and niacin in adolescents). For food groups (Table 4), irrespective of age, the NRF9.3 score was positively associated with breakfast intake of rice, potatoes, pulses, vegetables, fruits, eggs, dairy products (except for children), and seasonings (except for children) and inversely with that of bread, sugar, and soft drinks. There were also a positive association with fish and an inverse association with fats and oils in younger and older adults, as well as an inverse association with meat in adolescents and older adults.

## 4. Discussion

To our knowledge, this is the first study to evaluate breakfast intake in Japanese, using detailed dietary intake information from a national nutrition survey. Remarkably, despite the cultural differences in food intake patterns at breakfast between Western countries and Japan, there was a high degree of similarity in terms of regularity of breakfast consumption and contribution to overall nutrient intakes and diet quality. We found that the majority of participants (97% for whole sample) reported breakfast consumption on the dietary recording day. The figure is somewhat higher than those observed in other countries based on one-day dietary data, such as in the US (83% for children and 85% for adults) [6], Canada (overall 90%) [2], and Australia (91% for children) [7]. Our finding that the prevalence of breakfast skippers was high in young adults (7.7%) compared with other age groups (0.7–2.6%) might reflect their busy lifestyles (particularly working men). Further, the overall diet quality of breakfast consumers (as assessed by NRF9.3 score) was higher than that of breakfast skippers, which is very consistent with the findings from Western countries [1,2,6]. Additionally, the contribution of breakfast to daily energy intake (among breakfast consumers) was around 20–25% in this study. This is again highly consistent with the findings from IBRI project: 22% in Canada [2], 20–22% in the UK [3], 18% in France [4], 18–20% in Denmark [5], 19–22% in the US [6], and 16–19% in Spain [7]. This similarity appears interesting and warrants further investigation of the underlying reasons, particularly considering the large differences among countries in food choice and nutrient intake at breakfast, and in the definitions of breakfast and in dietary assessment methods.

In this study, the nutrient density of breakfast per se was on average low (except for older adults) compared with Western countries (particularly the US), where breakfast is much more nutrient-dense [2,3,4,5,6,7]. The reason for this relatively nutrient-poor breakfast in Japan is not precisely known, but may be due to high intake of refined grains as staple foods (such as rice and bread). In any case, while breakfast is of course only one meal and any shortfalls can be made up later in the day, these findings appear to suggest that there may be a room for optimizing breakfast consumption patterns in Japan.

As expected, and consistent with the findings from other countries in the IBRI project [2,3,4,5,6,7], the overall quality of daily diet assessed by the NRF9.3 score was positively associated with favorable profiles of breakfast intake. Irrespective of age, in the highest tertile of NRF9.3 score, the highest breakfast intake of many key nutrients was observed, as well as the lowest intake of added sugar. At the food group level, the highest breakfast intake of rice, potatoes, potatoes, pulses, vegetables, fruits, and eggs and the lowest intake of bread, sugar, and soft drinks were evident in the highest tertile of NRF9.3 score. As suggested by the IBRI statement [1], the pattern and intake level of breakfast in the highest tertile of NRF9.3 score would serve as valuable quantitative information for the development of dietary recommendation on a balanced breakfast in Japan.

Several limitations of this study should be mentioned. First, while the NHNSJ’s goal is for a nationally representative sample of non-institutionalized Japanese, participation was limited to only 52% of sampled households. Further, no information was available on the basic characteristics of households that refused to participate, and exact response rate was not determined at the individual level [21]. Moreover, the participants included in the present analysis (*n* = 22,452) differed somewhat from those excluded from the analysis because of missing information (*n* = 8038). The excluded participants were more likely to be male, be younger, and had lower daily energy intake. Additionally, because participants were recruited as members of households, the variability of dietary intake hay have decreased. Accordingly, although the dataset should provide a highly representative focus of dietary intake in Japan, there might exist the possibility of selection bias.

Second, self-reported dietary assessment is subject to random and systematic measurement errors [43,44,45]. Allowing that dietary intake varies daily among free-living individuals, data from our one-day weighed household dietary records are unlikely to represent the usual dietary intakes of individual respondents. Nevertheless, they are a reliable method to assess population averages. Further, the dietary intake was not done on proportionately selected days of the week. Moreover, Sundays were excluded, as specified in the survey protocol. This is also likely to have produced bias in the assessment of average dietary intakes. We have no data on which day was selected for recording [21]. Moreover, because the survey was conducted within a limited period of the year (from 25 October to 7 December 2012), the possibility of seasonal variation was also not considered. This may have also introduced bias into the assessment of average dietary intakes, especially considering the seasonal difference in food intakes among Japanese (e.g., higher fruit and lower vegetable intake in fall compared with other seasons) [46].

Additionally, self-reported dietary assessment suffers from the problem of misreporting of dietary intake, particularly in overweight and obese individuals [44,45], notwithstanding that all analyses were adjusted for energy intake. Of most importance, the use of this household dietary record in evaluating dietary intake in individuals has been validated in young women, but not for women of other age groups or men [27]. Assessment would have been preferable using several days, especially with the inclusion of all seasons and days of the week. Moreover, assessment would be improved by use of a validated dietary assessment questionnaire. The NHNSJ should evaluate whether these suggestions are feasible.

Finally, the definition of breakfast was based on self-report by nature. Although this appears to be the most widely used definition in breakfast research, self-defined breakfast may be subject to inconsistencies due to differences in individual perceptions [47]. Although we could not use another definition of breakfast (for example, based on time of day) because of the lack of information, different results may arise with different definitions. Investigation of this possibility will aid understanding of the nature and dietary and health impacts of breakfast.

## 5. Conclusions

Using one-day dietary data from a national nutrition survey in Japan, we found that the majority of participants (97%) reported breakfast consumption. The quality of the overall diet was higher in breakfast consumers than in skippers. Among breakfast consumers, 20–25% of daily energy intake came from breakfast, depending on age. Compared to its contribution to energy, breakfast contributed a higher proportion of total carbohydrate and riboflavin and a lower proportion of MUFA, *n*-3 PUFA, thiamin, niacin, and vitamins B-6 and C. In older adults, the contribution of breakfast to dietary fiber, vitamin K, folate, pantothenic acid, magnesium, and copper was higher than its contribution to energy, whereas that to added sugar was lower. Thus, the nutrient density of breakfast was on average insufficient, except for older adults. However, the overall quality of the daily diet assessed by the NRF9.3 score was associated positively with the breakfast intake of protein, *n*-6 PUFA, *n*-3 PUFA, carbohydrate, dietary fiber, and almost all micronutrients examined, and inversely with that of added sugar. For foods, the NRF9.3 score was positively associated with breakfast intake of rice, potatoes, pulses, vegetables, fruits, and eggs and inversely with bread, sugar, and soft drinks. These results suggest that consumption of balanced breakfast may be important for the improvement of overall diet quality. These findings should aid in the development of dietary recommendations for a balanced breakfast among Japanese populations.

## Figures and Tables

**Table 1:** Basic characteristics of breakfast skippers and consumers.

	Breakfast Skipper	Breakfast Consumer	*p* ^1^	Breakfast Skipper	Breakfast Consumer	*p* ^1^
**Age Group**	**Children (Aged 6–11 Years)**			**Adolescents (Aged 12–17 Years)**		
*n*	10	1434		30	1104	
Age (years), mean ± SD	8.2 ± 1.8	8.5 ± 1.7	0.56	15.3 ± 1.8	14.2 ± 1.7	0.001
Sex, *n* (%)			0.47			0.45
Male	6 (60)	695 (48.5)		18 (60)	585 (53.0)	
Female	4 (40)	739 (51.5)		12 (40)	519 (47.0)	
Weight status, *n* (%) ^2^			0.48			0.53
Underweight	0 (0)	37 (2.6)		1 (3.3)	21 (1.9)	
Normal weight	10 (100)	1250 (87.2)		25 (83.3)	990 (89.7)	
Overweight/obese	0 (0)	147 (10.3)		4 (13.3)	93 (8.4)	
**Age Group**	**Younger Adults (Aged 18–49 Years)**			**Older Adults (Aged ≥ 50 Years)**		
*n*	503	6028		208	13135	
Age (years), mean ± SD	34.3 ± 8.2	37.6 ± 7.5	<0.0001	62.2 ± 8.6	67.4 ± 9.8	<0.0001
Sex, *n* (%)			<0.0001			<0.0001
Male	313 (62.2)	2672 (44.3)		129 (62)	5672 (43.2)	
Female	190 (37.8)	3356 (55.7)		79 (38)	7463 (56.8)	
Weight status, *n* (%) ^3^			0.04			0.40
Underweight	53 (10.5)	624 (10.4)		9 (4.3)	836 (6.4)	
Normal weight	318 (63.2)	4111 (68.2)		138 (66.4)	8784 (66.9)	
Overweight/obese	132 (26.2)	1293 (21.5)		61 (29.3)	3515 (26.8)	
Occupation, *n* (%)			0.002			<0.0001
Professional/manager	104 (20.7)	1340 (22.2)		20 (9.6)	1299 (9.9)	
Sales/service/clerical	224 (44.5)	2363 (39.2)		45 (21.6)	2247 (17.1)	
Security/transportation/labor	119 (23.7)	1295 (21.5)		62 (29.8)	2405 (18.3)	
Not in paid employment	56 (11.1)	1030 (17.1)		81 (38.9)	7184 (54.7)	

^1^ Based on an independent *t*-test for age and chi-square test for other variables. ^2^ Defined according to the International Obesity Task Force on age- and sex-specific body mass index (BMI, in kg/m^2^) cutoffs, which correspond to an adult BMI < 17 for underweight, ≥17 to <25 for normal weight, and ≥25 for overweight/obese. ^3^ Defined based on BMI (kg/m^2^) according to World Health Organization recommendations: <18.5 for underweight, ≥18.5 to <25 for normal weight, and ≥25 for overweight/obese.

**Table 2:** Intakes of energy and macronutrients at breakfast among breakfast consumers according to tertile (T) category of the Nutrient-Rich Food Index 9.3 (NRF9.3) score as a measure of overall diet quality ^1^.

	Children (Aged 6–11 Years)			*p* for	Adolescents (Aged 12–17 Years)			*p* for	Younger Adults (Aged 18–49 Years)			*p* for	Older Adults (Aged ≥ 50 Years)			*p* for
	T1	T2	T3	Trend ^2^	T1	T2	T3	Trend ^2^	T1	T2	T3	Trend ^2^	T1	T2	T3	Trend ^2^
*n*	478	478	478		368	368	368		2009	2010	2009		4378	4379	4378	
NRF9.3 score, mean	570	703	780	-	507	651	752	-	447	616	728	-	537	685	787	
Energy (kcal)	367 ± 7	379 ± 7	375 ± 7	0.44	449 ± 10	453 ± 10	491 ± 10	0.003	377 ± 5	416 ± 5	442 ± 5	<0.0001	437 ± 3	471 ± 3	506 ± 3	<0.0001
Energy (% of all day)	20.1 ± 0.3	20.8 ± 0.3	21.5 ± 0.2	0.003	19.4 ± 0.4	19.8 ± 0.4	22.1 ± 0.4	<0.0001	19.4 ± 0.2	21.5 ± 0.2	22.7 ± 0.2	<0.0001	23.5 ± 0.1	24.7 ± 0.1	26.1 ± 0.1	<0.0001
Macronutrients (g)																
Protein	12.1 ± 0.3	13.2 ± 0.3	13.9 ± 0.3	<0.0001	14.5 ± 0.4	15.8 ± 0.4	17.5 ± 0.4	<0.0001	11.8 ± 0.2	14.3 ± 0.2	16.3 ± 0.2	<0.0001	15.6 ± 0.1	17.4 ± 0.1	19.4 ± 0.1	<0.0001
Fat	11.8 ± 0.4	12.1 ± 0.4	11.6 ± 0.4	0.58	13.3 ± 0.5	14.1 ± 0.4	14.5 ± 0.5	0.06	11.1 ± 0.2	12.3 ± 0.2	12.3 ± 0.2	<0.0001	11.3 ± 0.1	12.1 ± 0.1	13.0 ± 0.1	<0.0001
SFA	4.0 ± 0.1	4.0 ± 0.1	3.7 ± 0.1	0.11	4.2 ± 0.2	4.6 ± 0.2	4.5 ± 0.2	0.18	3.4 ± 0.1	3.7 ± 0.1	3.6 ± 0.1	0.09	3.3 ± 0.04	3.4 ± 0.04	3.8 ± 0.04	<0.0001
MUFA	4.0 ± 0.1	4.1 ± 0.1	3.9 ± 0.1	0.52	4.6 ± 0.2	4.7 ± 0.2	4.9 ± 0.2	0.23	3.8 ± 0.1	4.1 ± 0.1	4.0 ± 0.1	0.10	3.7 ± 0.1	3.9 ± 0.1	4.0 ± 0.1	<0.0001
*n*-6 PUFA	1.8 ± 0.1	1.9 ± 0.1	2.0 ± 0.1	0.01	2.1 ± 0.1	2.2 ± 0.1	2.6 ± 0.1	<0.0001	1.9 ± 0.04	2.3 ± 0.04	2.4 ± 0.04	<0.0001	2.2 ± 0.03	2.4 ± 0.03	2.7 ± 0.03	<0.0001
*n*-3 PUFA	0.27 ± 0.01	0.28 ± 0.01	0.32 ± 0.01	0.01	0.3 ± 0.02	0.4 ± 0.02	0.4 ± 0.02	0.0002	0.3 ± 0.01	0.4 ± 0.01	0.5 ± 0.01	<0.0001	0.4 ± 0.01	0.5 ± 0.01	0.6 ± 0.01	<0.0001
Carbohydrate	50.1 ± 0.9	52.2 ± 0.9	55.7 ± 0.9	<0.0001	63.5 ± 1.5	64.6 ± 1.4	73.9 ± 1.4	<0.0001	55.5 ± 0.7	61.0 ± 0.7	66.1 ± 0.7	<0.0001	68.8 ± 0.4	72.4 ± 0.4	76.2 ± 0.4	<0.0001
Added sugar	7.9 ± 0.3	4.4 ± 0.3	3.6 ± 0.3	<0.0001	6.8 ± 0.4	5.1 ± 0.4	4.1 ± 0.4	<0.0001	6.6 ± 0.2	4.5 ± 0.2	3.7 ± 0.2	<0.0001	6.5 ± 0.1	4.7 ± 0.1	4.0 ± 0.1	<0.0001
Dietary fiber	1.8 ± 0.1	2.2 ± 0.1	2.5 ± 0.1	<0.0001	2.0 ± 0.1	2.6 ± 0.1	3.3 ± 0.1	<0.0001	2.0 ± 0.1	2.8 ± 0.1	3.6 ± 0.1	<0.0001	3.4 ± 0.04	4.4 ± 0.04	5.3 ± 0.04	<0.0001
Macronutrient Balance (% Energy at Breakfast)																
Protein	13.3 ± 0.2	14.0 ± 0.2	14.3 ± 0.2	<0.0001	13.2 ± 0.2	14.1 ± 0.2	14.1 ± 0.2	0.005	12.8 ± 0.1	13.7 ± 0.1	14.5 ± 0.1	<0.0001	13.8 ± 0.1	14.6 ± 0.1	15.4 ± 0.1	<0.0001
Fat	28.0 ± 0.6	27.6 ± 0.6	25.6 ± 0.6	0.005	27.0 ± 0.7	26.8 ± 0.7	25.3 ± 0.7	0.07	24.0 ± 0.3	24.7 ± 0.3	23.8 ± 0.3	0.62	21.8 ± 0.2	22.0 ± 0.2	22.8 ± 0.2	0.0004
SFA	9.3 ± 0.3	9.0 ± 0.3	8.1 ± 0.3	0.0009	8.6 ± 0.3	8.8 ± 0.3	7.9 ± 0.3	0.09	7.7 ± 0.1	7.7 ± 0.1	7.2 ± 0.1	0.0009	6.4 ± 0.1	6.4 ± 0.1	6.7 ± 0.1	0.002
MUFA	9.4 ± 0.2	9.2 ± 0.2	8.4 ± 0.2	0.007	9.1 ± 0.3	8.7 ± 0.3	8.4 ± 0.3	0.07	7.9 ± 0.1	8.1 ± 0.1	7.6 ± 0.1	0.11	7.0 ± 0.1	7.0 ± 0.1	7.0 ± 0.1	0.89
PUFA	4.9 ± 0.1	5.0 ± 0.1	5.3 ± 0.1	0.06	4.8 ± 0.2	5.0 ± 0.2	5.3 ± 0.2	0.052	4.6 ± 0.1	5.2 ± 0.1	5.3 ± 0.1	<0.0001	5.0 ± 0.1	5.3 ± 0.1	5.6 ± 0.1	<0.0001
Carbohydrate	57.9 ± 0.7	57.4 ± 0.7	58.9 ± 0.7	0.28	58.5 ± 0.8	58.1 ± 0.8	59.5 ± 0.8	0.36	62.3 ± 0.4	60.8 ± 0.4	61.2 ± 0.4	0.02	63.6 ± 0.2	63.0 ± 0.2	61.9 ± 0.2	<0.0001
Added sugar	8.9 ± 0.4	4.8 ± 0.4	3.6 ± 0.4	<0.0001	7.2 ± 0.4	4.8 ± 0.4	3.4 ± 0.4	<0.0001	9.8 ± 0.3	5.4 ± 0.3	4.2 ± 0.3	<0.0001	6.6 ± 0.1	4.3 ± 0.1	3.4 ± 0.1	<0.0001

^1^ Values are means ± SEs unless otherwise indicated. Adjustment was made for age, sex, weight status, occupation (for younger and older adults only), and total energy intake (except for the analysis on energy intake itself). NRF9.3 score was calculated based on daily intake of nine nutrients to encourage (i.e., protein, dietary fiber, vitamins A, C, and D, calcium, iron, potassium, and magnesium) and three nutrients to limit (i.e., added sugar, saturated fat, and sodium). A higher score indicates a higher diet quality. ^2^ Calculated by using general linear models.

**Table 3:** Intakes of vitamins and minerals at breakfast among breakfast consumers according to tertile (T) category of the Nutrient-Rich Food Index 9.3 (NRF9.3) score as a measure of overall diet quality ^1^.

	Children (Aged 6–11 Years)			*p* for	Adolescents (Aged 12–17 Years)			*p* for	Younger adults (Aged 18–49 Years)			*p* for	Older Adults (Aged ≥ 50 Years)			*p* for
	T1	T2	T3	Trend ^2^	T1	T2	T3	Trend ^2^	T1	T2	T3	Trend ^2^	T1	T2	T3	Trend ^2^
*n*	478	478	478		368	368	368		2009	2010	2009		4378	4379	4378	
Vitamins																
Vitamin A (μg RAE)	70 ± 5	93 ± 5	109 ± 5	<0.0001	91 ± 15	101 ± 15	160 ± 15	0.002	59 ± 5	87 ± 5	145 ± 5	<0.0001	89 ± 5	142 ± 5	194 ± 5	<0.0001
Vitamin D (μg)	0.8 ± 0.1	0.8 ± 0.1	1.0 ± 0.1	0.07	0.9 ± 0.1	1.2 ± 0.1	1.5 ± 0.1	0.005	0.7 ± 0.1	1.3 ± 0.1	1.7 ± 0.1	<0.0001	1.6 ± 0.1	2.1 ± 0.1	2.5 ± 0.1	<0.0001
Vitamin E (mg)	1.0 ± 0.04	1.1 ± 0.04	1.2 ± 0.04	0.01	1.2 ± 0.1	1.3 ± 0.1	1.5 ± 0.1	0.0003	1.1 ± 0.03	1.4 ± 0.03	1.6 ± 0.03	<0.0001	1.4 ± 0.02	1.7 ± 0.02	2.1 ± 0.02	<0.0001
Vitamin K (μg)	29.6 ± 3.3	37.5 ± 3.3	53.0 ± 3.3	<0.0001	27.1 ± 4.3	41.8 ± 4.3	70.4 ± 4.3	<0.0001	29.8 ± 2.1	54.1 ± 2.1	81.9 ± 2.1	<0.0001	63.6 ± 1.8	90.5 ± 1.8	121 ± 1.8	<0.0001
Thiamin (mg)	0.14 ± 0.004	0.16 ± 0.004	0.17 ± 0.004	<0.0001	0.18 ± 0.01	0.20 ± 0.01	0.21 ± 0.01	0.007	0.14 ± 0.003	0.17 ± 0.003	0.20 ± 0.003	<0.0001	0.17 ± 0.002	0.20 ± 0.002	0.24 ± 0.002	<0.0001
Riboflavin (mg)	0.24 ± 0.01	0.27 ± 0.01	0.29 ± 0.01	<0.0001	0.26 ± 0.01	0.31 ± 0.01	0.36 ± 0.01	<0.0001	0.21 ± 0.004	0.27 ± 0.004	0.33 ± 0.004	<0.0001	0.30 ± 0.003	0.36 ± 0.003	0.43 ± 0.003	<0.0001
Niacin (mg)	1.8 ± 0.1	2.0 ± 0.1	2.2 ± 0.1	0.002	2.5 ± 0.1	2.5 ± 0.1	2.7 ± 0.1	0.20	2.3 ± 0.1	2.8 ± 0.1	3.2 ± 0.1	<0.0001	3.0 ± 0.04	3.5 ± 0.04	3.8 ± 0.04	<0.0001
Vitamin B-6 (mg)	0.14 ± 0.01	0.17 ± 0.01	0.20 ± 0.01	<0.0001	0.17 ± 0.01	0.21 ± 0.01	0.26 ± 0.01	<0.0001	0.15 ± 0.004	0.21 ± 0.004	0.28 ± 0.004	<0.0001	0.25 ± 0.003	0.32 ± 0.003	0.39 ± 0.003	<0.0001
Vitamin B-12 (μg)	0.8 ± 0.1	0.9 ± 0.1	0.9 ± 0.1	0.48	1.0 ± 0.1	1.1 ± 0.1	1.2 ± 0.1	0.15	0.6 ± 0.01	0.9 ± 0.01	1.2 ± 0.01	<0.0001	1.3 ± 0.1	1.5 ± 0.1	1.8 ± 0.1	<0.0001
Folate (μg)	42.0 ± 1.6	47.8 ± 1.6	58.6 ± 1.6	<0.0001	48.4 ± 2.6	58.6 ± 2.5	80.5 ± 2.6	<0.0001	44.5 ± 1.2	64.0 ± 1.2	87.9 ± 1.2	<0.0001	79.1 ± 1.0	106 ± 1.0	128 ± 1.0	<0.0001
Pantothenic acid (mg)	1.1 ± 0.03	1.3 ± 0.03	1.4 ± 0.03	<0.0001	1.3 ± 0.04	1.4 ± 0.04	1.8 ± 0.04	<0.0001	1.0 ± 0.02	1.3 ± 0.02	1.6 ± 0.02	<0.0001	1.4 ± 0.01	1.7 ± 0.01	2.1 ± 0.01	<0.0001
Vitamin C (mg)	13.3 ± 1.3	15.1 ± 1.3	18.5 ± 1.3	0.007	14.7 ± 1.2	16.6 ± 1.2	23.7 ± 1.2	<0.0001	10.9 ± 0.7	17.8 ± 0.7	26.5 ± 0.7	<0.0001	23.6 ± 0.5	35.1 ± 0.5	44.1 ± 0.5	<0.0001
Minerals																
Sodium (g NaCl equivalent)	1.7 ± 0.05	1.9 ± 0.05	1.7 ± 0.05	0.33	2.1 ± 0.07	2.2 ± 0.07	2.3 ± 0.07	0.06	1.8 ± 0.03	2.1 ± 0.03	2.1 ± 0.03	<0.0001	2.8 ± 0.03	2.9 ± 0.03	2.7 ± 0.03	0.02
Potassium (mg)	349 ± 11	404 ± 11	458 ± 11	<0.0001	389 ± 15	481 ± 15	589 ± 15	<0.0001	363 ± 7	487 ± 7	628 ± 7	<0.0001	578 ± 6	736 ± 6	900 ± 6	<0.0001
Calcium (mg)	118 ± 5	132 ± 5	134 ± 5	0.03	117 ± 6	157 ± 6	165 ± 6	<0.0001	96 ± 2	124 ± 2	151 ± 2	<0.0001	137 ± 2	171 ± 2	221 ± 2	<0.0001
Magnesium (mg)	36.8 ± 1.1	42.2 ± 1.1	48.6 ± 1.1	<0.0001	41.7 ± 1.5	53.1 ± 1.5	62.5 ± 1.5	<0.0001	41.2 ± 0.7	53.8 ± 0.7	66.3 ± 0.7	<0.0001	63.5 ± 0.6	76.7 ± 0.6	90.3 ± 0.6	<0.0001
Phosphorus (mg)	196 ± 5	218 ± 5	226 ± 5	<0.0001	225 ± 7	256 ± 6	282 ± 7	<0.0001	179 ± 3	224 ± 3	261 ± 3	<0.0001	243 ± 2	283 ± 2	328 ± 2	<0.0001
Iron (mg)	1.1 ± 0.04	1.3 ± 0.04	1.5 ± 0.04	<0.0001	1.3 ± 0.05	1.5 ± 0.05	1.9 ± 0.05	<0.0001	1.2 ± 0.03	1.6 ± 0.03	2.0 ± 0.03	<0.0001	1.9 ± 0.02	2.3 ± 0.02	2.7 ± 0.02	<0.0001
Zinc (mg)	1.4 ± 0.03	1.6 ± 0.03	1.7 ± 0.03	<0.0001	1.8 ± 0.05	1.9 ± 0.05	2.2 ± 0.05	<0.0001	1.4 ± 0.02	1.7 ± 0.02	2.0 ± 0.02	<0.0001	1.9 ± 0.01	2.1 ± 0.01	2.3 ± 0.01	<0.0001
Copper (mg)	0.18 ± 0.005	0.20 ± 0.006	0.24 ± 0.006	<0.0001	0.23 ± 0.008	0.26 ± 0.008	0.32 ± 0.008	<0.0001	0.19 ± 0.004	0.25 ± 0.004	0.31 ± 0.004	<0.0001	0.30 ± 0.003	0.35 ± 0.003	0.40 ± 0.003	<0.0001
Manganese (mg)	0.4 ± 0.01	0.5 ± 0.01	0.6 ± 0.01	<0.0001	0.6 ± 0.02	0.7 ± 0.02	0.8 ± 0.02	<0.0001	0.5 ± 0.01	0.7 ± 0.01	0.8 ± 0.01	<0.0001	0.9 ± 0.01	1.0 ± 0.01	1.1 ± 0.01	<0.0001

RAE, retinol activity equivalent. ^1^ Values are means ± SEs unless otherwise indicated. Adjustment was made for age, sex, weight status, occupation (for younger and older adults only), and total energy intake. NRF9.3 score was calculated based on daily intake of nine nutrients to encourage (i.e., protein, dietary fiber, vitamins A, C, and D, calcium, iron, potassium, and magnesium) and three nutrients to limit (i.e., added sugar, saturated fat, and sodium). A higher score indicates a higher diet quality. ^2^ Calculated by using general linear models.

**Table 4:** Intakes of food groups at breakfast (in grams) among breakfast consumers according to tertile (T) category of the Nutrient-Rich Food Index 9.3 (NRF9.3) score as a measure of overall diet quality ^1^.

	Children (Aged 6–11 Years)			*p* for	Adolescents (Aged 12–17 Years)			*p* for	Younger Adults (Aged 18–49 Years)			*p* for	Older Adults (Aged ≥ 50 years)			*p* for
	T1	T2	T3	Trend ^2^	T1	T2	T3	Trend ^2^	T1	T2	T3	Trend ^2^	T1	T2	T3	Trend ^2^
n	478	478	478		368	368	368		2009	2010	2009		4378	4379	4378	
Rice	50.6 ± 2.9 (45%)	64.0 ± 2.9 (57%)	78.4 ± 2.9 (68%)	<0.0001	87.3 ± 5.0 (51%)	84.1 ± 5.0 (54%)	107.8 ± 5.0 (67%)	0.004	62.0 ± 2.0 (37%)	78.2 ± 2.0 (48%)	91.7 ± 2.0 (57%)	<0.0001	88.0 ± 1.3 (55%)	97.1 ± 1.3 (62%)	95.8 ± 1.3 (62%)	<0.0001
Bread	22.0 ± 1.4 (38%)	20.5 ± 1.4 (36%)	14.1 ± 1.4 (27%)	<0.0001	22.8 ± 1.9 (34%)	25.1 ± 1.9 (37%)	18.5 ± 1.9 (27%)	0.10	27.1 ± 0.8 (38%)	24.6 ± 0.8 (35%)	20.2 ± 0.8 (30%)	<0.0001	23.4 ± 0.5 (34%)	21.3 ± 0.5 (32%)	20.1 ± 0.5 (30%)	<0.0001
Other grains	3.7 ± 0.6 (11%)	3.1 ± 0.6 (12%)	2.7 ± 0.6 (10%)	0.26	4.7 ± 0.8 (12%)	2.3 ± 0.8 (10%)	3.0 ± 0.8 (9%)	0.17	2.8 ± 0.3 (8%)	2.0 ± 0.3 (7%)	2.1 ± 0.3 (8%)	0.09	1.9 ± 0.2 (7%)	1.6 ± 0.2 (6%)	2.3 ± 0.2 (7%)	0.14
Potatoes	2.7 ± 0.7 (13%)	5.4 ± 0.7 (18%)	7.0 ± 0.7 (24%)	<0.0001	3.3 ± 0.8 (13%)	4.3 ± 0.8 (14%)	6.1 ± 0.8 (16%)	0.02	3.0 ± 0.4 (10%)	5.4 ± 0.4 (14%)	7.7 ± 0.4 (18%)	<0.0001	7.5 ± 0.4 (16%)	10.1 ± 0.4 (22%)	11.5 ± 0.4 (24%)	<0.0001
Sugar	2.4 ± 0.2 (30%)	1.5 ± 0.2 (29%)	1.1 ± 0.2 (24%)	<0.0001	2.3 ± 0.3 (31%)	2.2 ± 0.3 (33%)	1.5 ± 0.3 (30%)	0.049	2.4 ± 0.1 (31%)	2.0 ± 0.1 (30%)	1.8 ± 0.1 (31%)	<0.0001	3.5 ± 0.1 (42%)	2.8 ± 0.1 (40%)	2.4 ± 0.1 (39%)	<0.0001
Pulses	7.7 ± 0.9 (23%)	8.1 ± 0.9 (27%)	12.6 ± 0.9 (37%)	0.0001	7.8 ± 1.4 (22%)	12.6 ± 1.4 (35%)	18.2 ± 1.4 (42%)	<0.0001	8.6 ± 0.8 (18%)	16.6 ± 0.8 (32%)	22.2 ± 0.8 (42%)	<0.0001	18.9 ± 0.6 (39%)	25.0 ± 0.6 (49%)	31.1 ± 0.6 (58%)	<0.0001
Vegetables	16.2 ± 1.6 (49%)	26 ± 1.6 (64%)	30.4 ± 1.6 (68%)	<0.0001	23.1 ± 2.2 (55%)	28.8 ± 2.2 (62%)	44.1 ± 2.2 (73%)	<0.0001	19.6 ± 1.2 (43%)	35 ± 1.2 (57%)	54.4 ± 1.2 (69%)	<0.0001	48.4 ± 1.1 (68%)	74.4 ± 1.1 (78%)	92.0 ± 1.1 (83%)	<0.0001
Fruits	12.4 ± 1.7 (21%)	16.9 ± 1.7 (26%)	21.2 ± 1.7 (31%)	0.0003	11.1 ± 2.4 (18%)	21.6 ± 2.4 (27%)	28.4 ± 2.4 (34%)	<0.0001	12.8 ± 1.0 (17%)	18.8 ± 1.0 (23%)	26.6 ± 1.0 (32%)	<0.0001	26.4 ± 1.0 (31%)	40.9 ± 1.0 (44%)	55.9 ± 1.0 (54%)	<0.0001
Fish	3.1 ± 0.6 (15%)	3.8 ± 0.6 (18%)	4.5 ± 0.6 (20%)	0.07	6.0 ± 0.9 (18%)	5.8 ± 0.9 (20%)	5.1 ± 0.9 (21%)	0.48	4.6 ± 0.5 (15%)	6.3 ± 0.5 (19%)	8.9 ± 0.5 (26%)	<0.0001	9.9 ± 0.4 (26%)	12.4 ± 0.4 (35%)	13.5 ± 0.4 (40%)	<0.0001
Meat	12.7 ± 0.9 (42%)	13.1 ± 0.9 (46%)	11.5 ± 0.9 (42%)	0.38	17.4 ± 1.2 (46%)	14.1 ± 1.2 (45%)	14.0 ± 1.2 (46%)	0.04	9.6 ± 0.5 (27%)	10.1 ± 0.5 (31%)	9.4 ± 0.5 (32%)	0.82	6.5 ± 0.2 (22%)	6.7 ± 0.2 (24%)	5.5 ± 0.2 (20%)	0.003
Eggs	11.7 ± 0.9 (36%)	14.1 ± 0.9 (44%)	16.1 ± 0.9 (47%)	0.0008	13.5 ± 1.2 (38%)	14.0 ± 1.2 (39%)	18.1 ± 1.2 (48%)	0.005	9.3 ± 0.5 (25%)	12.1 ± 0.5 (32%)	14.9 ± 0.5 (37%)	<0.0001	13 ± 0.3 (31%)	14.2 ± 0.3 (34%)	15.6 ± 0.3 (37%)	<0.0001
Dairy products	56.1 ± 3.9 (44%)	65.4 ± 4.0 (46%)	57.0 ± 3.9 (46%)	0.87	50.2 ± 4.9 (42%)	72.4 ± 4.8 (49%)	71.8 ± 4.8 (50%)	0.002	35.9 ± 1.8 (39%)	44.9 ± 1.8 (42%)	53.0 ± 1.8 (43%)	<0.0001	40.1 ± 1.3 (39%)	52.0 ± 1.3 (43%)	75.0 ± 1.3 (52%)	<0.0001
Fats and oils	2.0 ± 0.1 (50%)	1.8 ± 0.1 (51%)	1.6 ± 0.1 (50%)	0.12	2.6 ± 0.2 (53%)	2.0 ± 0.2 (52%)	2.2 ± 0.2 (55%)	0.10	2.5 ± 0.1 (43%)	2.4 ± 0.1 (45%)	1.8 ± 0.1 (43%)	<0.0001	2.2 ± 0.1 (42%)	1.9 ± 0.1 (41%)	1.5 ± 0.1 (39%)	<0.0001
Soft drinks	21.3 ± 1.8 (20%)	7.9 ± 1.8 (10%)	4.7 ± 1.8 (11%)	<0.0001	11.5 ± 1.8 (10%)	7.8 ± 1.8 (11%)	3.8 ± 1.8 (8%)	0.002	22.0 ± 1.2 (12%)	10.2 ± 1.2 (8%)	5.9 ± 1.2 (7%)	<0.0001	9.6 ± 0.5 (8%)	6.3 ± 0.5 (7%)	5.3 ± 0.5 (7%)	<0.0001
Tea and coffee	38.6 ± 3.2 (28%)	41.1 ± 3.8 (30%)	43.4 ± 3.2 (34%)	0.28	51.9 ± 4.7 (33%)	62.8 ± 4.6 (39%)	63.0 ± 4.6 (39%)	0.10	121.9 ± 2.9 (61%)	116.9 ± 2.9 (59%)	119.2 ± 2.9 (61%)	0.51	137.4 ± 2.1 (67%)	148.8 ± 2.1 (69%)	149.8 ± 2.1 (68%)	<0.0001
Seasonings	9.6 ± 0.9 (68%)	9.6 ± 0.9 (78%)	9.9 ± 0.9 (79%)	0.83	10.5 ± 1.4 (69%)	13.3 ± 1.4 (72%)	14.7 ± 1.4 (80%)	0.03	9.3 ± 0.6 (53%)	13.0 ± 0.6 (66%)	14.1 ± 0.6 (74%)	<0.0001	15.6 ± 0.5 (72%)	18.4 ± 0.5 (80%)	19.9 ± 0.5 (82%)	<0.0001

^1^ Values are means ± SEs (percentages of consumers) unless otherwise indicated. Adjustment was made for age, sex, weight status, occupation (for younger and older adults only), and total energy intake. NRF9.3 score was calculated as a measure of overall diet quality, based on daily intake of nine nutrients to encourage (i.e., protein, dietary fiber, vitamins A, C, and D, calcium, iron, potassium, and magnesium) and three nutrients to limit (i.e., added sugar, saturated fat, and sodium). A higher score indicates a higher diet quality. Food groups with <10% consumers at breakfast in all populations (i.e., noodles, nuts, confectioneries, fruit juice, and vegetable juice) were not investigated. ^2^ Calculated by using general linear models.

## Supplementary Materials

The following are available online at http://www.mdpi.com/2072-6643/10/10/1551/s1, Table S1: Age- and sex-specific reference daily values used for the calculation of the Nutrient-Rich Food Index 9.3 (NRF9.3) score; Table S2: Basic characteristics of participants according to tertile (T) category of the Nutrient-Rich Food Index 9.3 (NRF9.3) score as a measure of overall diet quality; Table S3: Daily intakes of energy and macronutrients according to tertile (T) category of the Nutrient-Rich Food Index 9.3 (NRF9.3) score as a measure of overall diet quality; Table S4: Daily intakes of vitamins and minerals according to tertile (T) category of the Nutrient-Rich Food Index 9.3 (NRF9.3) score as a measure of overall diet quality; Table S5: Daily intakes of food groups (in grams) according to tertile (T) category of the Nutrient-Rich Food Index 9.3 (NRF9.3) score as a measure of overall diet quality; Figure S1: Proportion of breakfast skippers and consumers. Total *n* = 1444 for children, 1144 for adolescents, 6531 for younger adults, and 13,343 for older adults; Table S6: Daily intakes of breakfast skippers and consumers: Nutrient-Rich Food Index 9.3 (NRF9.3) score, energy, and macronutrients; Table S7: Daily intakes of breakfast skippers and consumers: vitamins and minerals; Table S8: Daily intakes of breakfast skippers and consumers: food groups (in grams); Table S9: Intakes of food groups (in grams) and percentage of consumers at breakfast among breakfast consumers; Table S10: Intakes of energy, macronutrients, vitamins, and minerals at breakfast among breakfast consumers.
