# Supplementary material for: Breakfast in Japan: Findings from the 2012 National Health and Nutrition Survey
Source: Nutrients. 2018 Oct 19;10(10):1551. doi: 10.3390/nu10101551 (PMC6212985; doi:10.3390/nu10101551)
Supplement: Supplementary file 1 [file nutrients-10-01551-s001.pdf]

Table S1. Age- and sex-specific reference daily values used for the calculation of the Nutrient-Rich Food Index 9.3 (NRF9.3) score [1].

Age Category (years)	6–7		8–9		10–11		12–14		15–17		18–29		30–49		50–69		≥70	
**Sex**	**M**	**F**	**M**	**F**	**M**	**F**	**M**	**F**	**M**	**F**	**M**	**F**	**M**	**F**	**M**	**F**	**M**	**F**
Energy (kcal)[2]	1550	1450	1850	1700	2250	2100	2600	2400	2850	2300	2650	1950	2650	2000	2450	1900	2200	1750
**Qualifying nutrients**																		
Protein (g)[3]	35	30	40	40	50	50	60	55	65	55	60	50	60	50	60	50	60	50
Dietary fiber (g)[4]	11	10	12	12	13	13	17	16	19	17	20	18	20	18	20	18	19	17
Vitamin A (μg RAE)[3]	450	400	500	500	600	600	800	700	900	650	850	650	900	700	850	700	800	650
Vitamin C (mg)[3]	55	55	60	60	75	75	95	95	100	100	100	100	100	100	100	100	100	100
Vitamin D (μg)[5]	3	3	3.5	3.5	4.5	4.5	5.5	5.5	6	6	5.5	5.5	5.5	5.5	5.5	5.5	5.5	5.5
Calcium (mg)[3]	600	550	650	750	700	750	1000	800	800	650	800	650	650	650	700	650	700	650
Iron (mg)[3]	6.5	6.5	8	8.5	10	14	11.5	14	9.5	10.5	7	10.5	7.5	10.5	7.5	6.5	7	6
Potassium (mg)[4]	1800	1800	2000	2000	2200	2000	2600	2400	3000	2600	3000	2600	3000	2600	3000	2600	3000	2600
Magnesium (mg)[3]	130	130	170	160	210	220	290	290	360	310	340	270	370	290	350	290	320	270
**Disqualifying nutrients**																		
Added sugar (g)[6]	19.4	18.1	23.1	21.3	28.1	26.3	32.5	30.0	35.6	28.8	33.1	24.4	33.1	25.0	30.6	23.8	27.5	21.9
Saturated fat (g)[7]	12.1	11.3	14.4	13.2	17.5	16.3	20.2	18.7	22.2	17.9	20.6	15.2	20.6	15.6	19.1	14.8	17.1	13.6
Sodium (g NaCl equivalent)[4]	5	5.5	5.5	6	6.5	7	8	7	8	7	8	7	8	7	8	7	8	7

F, female; M, male; RAE, retinol activity equivalent. [1] Values were derived from the Dietary Reference Intakes for Japanese, 2015 except for added sugar (as shown below). [2] Estimated Energy Requirement for moderate level of physical activity. [3] Recommended Dietary Allowance. [4] Tentative dietary goal for preventing lifestyle-related diseases (DG). [5] Adequate Intake. [6] Determined based on the World Health Organization's conditional recommendation (5% of energy). [7] Determined based on the DG value (7% of energy).

Table S2. Basic characteristics of participants according to tertile (T) category of the Nutrient-Rich Food Index 9.3 (NRF9.3) score as a measure of overall diet quality[1].

	T1	T2	T3	p for trend [2]	T1	T2	T3	p for trend [2]
**Age Group**	**Children (aged 6–11 years)**				**Adolescents (aged 12–17 years)**			
n	**481**	**482**	**481**		**378**	**378**	**378**	
NRF9.3 score	568 ± 93	702 ± 22	780 ± 32	---	502 ± 105	649 ± 28	752 ± 38	---
Age (years)	8.4 ± 1.7	8.7 ± 1.7	8.4 ± 1.7	0.03	14.6 ± 1.7	14.1 ± 1.7	14.1 ± 1.6	<0.0001
Sex, n (%)				0.44				0.06
Male	231 (48.0)	227 (47.1)	243 (50.5)		188 (49.7)	201 (53.2)	214 (56.6)	
Female	250 (52.0)	255 (52.9)	238 (49.5)		190 (50.3)	177 (46.8)	164 (43.4)	
Weight status, n (%)[3]				0.46				
Underweight	13 (2.7)	10 (2.1)	14 (2.9)		9 (2.4)	6 (1.6)	7 (1.9)	0.57
Normal weight	423 (87.9)	408 (84.7)	429 (89.2)		334 (88.4)	338 (89.4)	343 (90.7)	
Overweight/obese	45 (9.4)	64 (13.3)	38 (7.9)		35 (9.3)	34 (9.0)	28 (7.4)	
**Age group**	**Younger Adults (aged 18–49 years)**				**Older adults (aged ≥50 years)**			
n	2177	2177	2177		4447	4448	4448	
NRF9.3 score	437 ± 107	611 ± 33	732 ± 47	---	534 ± 92	684 ± 28	786 ± 38	---
Age (years)	36.5 ± 7.8	37.7 ± 7.5	37.9 ± 7.4	<0.0001	66.0 ± 10.3	67.3 ± 9.6	68.5 ± 9.2	<0.0001
Sex, n (%)				<0.0001				0.01
Male	897 (41.2)	1033 (47.5)	1055 (48.5)		1990 (44.8)	1940 (43.6)	1871 (42.1)	
Female	1280 (58.8)	1144 (52.6)	1122 (51.5)		2457 (55.3)	2508 (56.4)	2577 (57.9)	
Weight status, n (%)[4]				0.81				0.0002
Underweight	250 (11.5)	204 (9.4)	223 (10.2)		274 (6.2)	284 (6.4)	287 (6.5)	
Normal weight	1443 (66.3)	1498 (68.8)	1488 (68.4)		2882 (64.8)	2996 (67.4)	3044 (68.4)	
Overweight/obese	484 (22.2)	475 (21.8)	466 (21.4)		1291 (29.0)	1168 (26.3)	1117 (25.1)	
Occupation, n (%)				<0.0001				<0.0001
Professional/manager	393 (18.1)	468 (21.5)	583 (26.8)		416 (9.4)	442 (9.9)	461 (10.4)	
Sales/service/clerical	879 (40.4)	871 (40.0)	837 (38.5)		894 (20.1)	792 (17.8)	606 (13.6)	
Security/transportation/labor	507 (23.3)	468 (21.5)	439 (20.2)		907 (20.4)	807 (18.1)	753 (16.9)	
Not in paid employment	398 (18.3)	370 (17)	318 (14.6)		2230 (50.2)	2407 (54.1)	2628 (59.1)	

[1] Values are means ± SDs unless otherwise indicated. NRF9.3 score was calculated based on daily intake of nine nutrients to encourage (i.e., protein, dietary fiber, vitamins A, C, and D, calcium, iron, potassium, and magnesium) and three nutrients to limit (i.e., added sugar, saturated fat, and sodium). A higher score indicates a higher diet quality. [2] Calculated by using general linear models for continuous variables and a Mantel-Haenszel chi-square test for categorical variables. [3] Defined according to the International Obesity Task Force on age- and sex-specific body mass index (BMI, in kg/m^2) cutoffs, which correspond to an adult BMI <17 for underweight, ≥17 to <25 for normal weight, and ≥25 for overweight/obese. [4] Defined based on BMI (kg/m^2) according to World Health Organization recommendations: <18.5 for underweight, ≥18.5 to <25 for normal weight, and ≥25 for overweight/obese.

Table S3. Daily intakes of energy and macronutrients according to tertile (T) category of the Nutrient-Rich Food Index 9.3 (NRF9.3) score as a measure of overall diet quality [1].

	**Children (aged 6–11 years)**			**p for Trend [2]**	**Adolescents (aged 12–17 years)**			**p for trend[2]**	**Younger adults (aged 18–49 years)**			**p for Trend [2]**	**Older adults (aged ≥ 50 years)**			**p for Trend [2]**
	**T1**	**T2**	**T3**		**T1**	**T2**	**T3**		**T1**	**T2**	**T3**		**T1**	**T2**	**T3**	
n	**481**	**482**	**481**		**378**	**378**	**378**		**2177**	**2177**	**2177**		**4447**	**4448**	**4448**	
Energy (kcal)	1841 ± 17	1816 ± 17	1739 ± 17	<0.0001	2350 ± 31	2299 ± 30	2228 ± 30	0.005	1952 ± 12	1955 ± 12	1945 ± 12	0.67	1894 ± 7	1936 ± 7	1962 ± 7	<0.0001
**Macronutrients (g)**																
Protein	63.0 ± 0.5	66.0 ± 0.5	68.6 ± 0.5	<0.0001	77.0 ± 0.7	83.6 ± 0.7	86.7 ± 0.7	<0.0001	63.5 ± 0.3	70.5 ± 0.3	74.6 ± 0.3	<0.0001	67.3 ± 0.2	72.6 ± 0.2	76.9 ± 0.2	<0.0001
Fat	61.5 ± 0.5	60.5 ± 0.5	57.0 ± 0.5	<0.0001	77.0 ± 0.9	76.1 ± 0.9	71.8 ± 0.9	<0.0001	61.6 ± 0.4	60.9 ± 0.3	58.5 ± 0.4	<0.0001	53.2 ± 0.2	52.8 ± 0.2	52.4 ± 0.2	0.04
SFA	21.5 ± 0.2	20.2 ± 0.2	18.6 ± 0.2	<0.0001	24.4 ± 0.3	24.1 ± 0.3	21.9 ± 0.3	<0.0001	18.4 ± 0.1	17.3 ± 0.1	15.8 ± 0.1	<0.0001	15.1 ± 0.1	14.3 ± 0.1	13.8 ± 0.1	<0.0001
MUFA	21.7 ± 0.2	20.8 ± 0.2	19.1 ± 0.2	<0.0001	28.8 ± 0.4	27.4 ± 0.4	25.4 ± 0.4	<0.0001	23.5 ± 0.2	22.7 ± 0.2	21.3 ± 0.2	<0.0001	19.4 ± 0.1	18.7 ± 0.1	18.1 ± 0.1	<0.0001
n-6 PUFA	9.0 ± 0.1	9.5 ± 0.1	9.5 ± 0.1	0.007	12.2 ± 0.2	12.3 ± 0.2	12.2 ± 0.2	0.94	10.4 ± 0.1	11.0 ± 0.1	10.9 ± 0.1	<0.0001	9.5 ± 0.1	9.8 ± 0.1	9.9 ± 0.1	<0.0001
n-3 PUFA	1.6 ± 0.04	1.9 ± 0.04	2.0 ± 0.04	<0.0001	2.1 ± 0.1	2.4 ± 0.1	2.6 ± 0.1	<0.0001	1.8 ± 0.03	2.3 ± 0.03	2.6 ± 0.03	<0.0001	2.2 ± 0.02	2.6 ± 0.02	2.9 ± 0.02	<0.0001
Carbohydrate	243 ± 1	241 ± 1	247 ± 1	0.01	312 ± 2	308 ± 2	316 ± 2	0.16	262 ± 1	261 ± 1	266 ± 1	0.0004	273 ± 1	273 ± 1	273 ± 1	0.91
Added sugar	39.1 ± 0.8	22.4 ± 0.8	18.0 ± 0.8	<0.0001	43.6 ± 1.1	25.9 ± 1.1	21.6 ± 1.1	<0.0001	35.8 ± 0.4	22.4 ± 0.4	18.0 ± 0.4	<0.0001	33.8 ± 0.3	23.6 ± 0.3	18.1 ± 0.3	<0.0001
Dietary fiber	10.4 ± 0.1	12.2 ± 0.1	13.4 ± 0.1	<0.0001	11.8 ± 0.2	14.0 ± 0.2	16.5 ± 0.2	<0.0001	10.3 ± 0.1	12.8 ± 0.1	15.8 ± 0.1	<0.0001	13.3 ± 0.1	16.5 ± 0.1	19.3 ± 0.1	<0.0001
**Macronutrient balance (% energy)**																
Protein	14.0 ± 0.1	14.8 ± 0.1	15.4 ± 0.1	<0.0001	13.5 ± 0.1	14.7 ± 0.1	15.4 ± 0.1	<0.0001	13.2 ± 0.1	14.6 ± 0.1	15.4 ± 0.1	<0.0001	14.1 ± 0.04	15.2 ± 0.04	16.0 ± 0.04	<0.0001
Fat	30.6 ± 0.3	30.1 ± 0.3	28.1 ± 0.3	<0.0001	30.3 ± 0.3	29.6 ± 0.3	27.9 ± 0.3	<0.0001	28.1 ± 0.2	27.9 ± 0.2	26.8 ± 0.2	<0.0001	24.3 ± 0.1	24.4 ± 0.1	24.4 ± 0.1	0.74
SFA	10.6 ± 0.1	10.1 ± 0.1	9.2 ± 0.1	<0.0001	9.6 ± 0.1	9.3 ± 0.1	8.5 ± 0.1	<0.0001	8.4 ± 0.1	7.9 ± 0.1	7.2 ± 0.1	<0.0001	6.8 ± 0.04	6.6 ± 0.04	6.4 ± 0.04	<0.0001
MUFA	10.8 ± 0.1	10.3 ± 0.1	9.4 ± 0.1	<0.0001	11.3 ± 0.1	10.6 ± 0.1	9.8 ± 0.1	<0.0001	10.6 ± 0.1	10.3 ± 0.1	9.7 ± 0.1	<0.0001	8.8 ± 0.1	8.6 ± 0.1	8.4 ± 0.1	<0.0001
PUFA	5.3 ± 0.1	5.6 ± 0.1	5.7 ± 0.1	0.0002	5.6 ± 0.1	5.7 ± 0.1	5.8 ± 0.1	0.09	5.6 ± 0.04	6.1 ± 0.04	6.2 ± 0.04	<0.0001	5.4 ± 0.03	5.8 ± 0.03	6.0 ± 0.03	<0.0001
Carbohydrate	54.0 ± 0.3	53.8 ± 0.3	55.3 ± 0.3	0.003	54.3 ± 0.4	54.0 ± 0.4	55.2 ± 0.4	0.08	54.2 ± 0.2	53.8 ± 0.2	54.7 ± 0.2	0.0499	57.4 ± 0.1	57 ± 0.1	56.9 ± 0.1	0.005
Added sugar	8.6 ± 0.2	5.0 ± 0.2	3.8 ± 0.2	<0.0001	7.5 ± 0.2	4.5 ± 0.2	3.6 ± 0.2	<0.0001	7.3 ± 0.1	4.5 ± 0.1	3.7 ± 0.1	<0.0001	6.9 ± 0.1	4.8 ± 0.1	3.8 ± 0.1	<0.0001

[1] Values are means ± SEs unless otherwise indicated. Adjustment was made for age, sex, weight status, occupation (for younger and older adults only), and total energy intake (except for the analysis on energy intake itself). NRF9.3 score was calculated based on daily intake of nine nutrients to encourage (i.e., protein, dietary fiber, vitamins A, C, and D, calcium, iron, potassium, and magnesium) and three nutrients to limit (i.e., added sugar, saturated fat, and sodium). A higher score indicates a higher diet quality. [2] Calculated by using general linear models.

Table S4. Daily intakes of vitamins and minerals according to tertile (T) category of the Nutrient-Rich Food Index 9.3 (NRF9.3) score as a measure of overall diet quality [1].

	Children (aged 6–11 years)			p for	Adolescents (aged 12–17 years)			p for	Younger adults (aged 18–49 years)			p for	Older adults (aged ≥ 50 years)			p for
	T1	T2	T3	Trend [2]	T1	T2	T3	Trend [2]	T1	T2	T3	Trend [2]	T1	T2	T3	Trend [2]
n	**481**	**482**	**481**		**378**	**378**	**378**		**2177**	**2177**	**2177**		**4447**	**4448**	**4448**	
**Vitamins**																
Vitamin A (μg RAE)	451 ± 14	538 ± 14	588 ± 14	<0.0001	473 ± 30	619 ± 30	754 ± 30	<0.0001	330 ± 16	486 ± 16	699 ± 16	<0.0001	395 ± 13	585 ± 13	806 ± 13	<0.0001
Vitamin D (μg)	4.4 ± 0.3	6.4 ± 0.3	7.2 ± 0.3	<0.0001	5.2 ± 0.4	7.0 ± 0.4	9.7 ± 0.4	<0.0001	3.9 ± 0.2	6.6 ± 0.2	9.0 ± 0.2	<0.0001	6.7 ± 0.1	9.3 ± 0.1	11.9 ± 0.1	<0.0001
Vitamin E (mg)	5.6 ± 0.1	6.1 ± 0.1	6.4 ± 0.1	<0.0001	6.9 ± 0.1	7.4 ± 0.1	8.1 ± 0.1	<0.0001	5.7 ± 0.1	6.6 ± 0.1	7.7 ± 0.1	<0.0001	6.1 ± 0.04	7.2 ± 0.04	8.4 ± 0.04	<0.0001
Vitamin K (μg)	144 ± 5	183 ± 5	212 ± 5	<0.0001	174 ± 7	212 ± 7	288 ± 7	<0.0001	147 ± 3	217 ± 3	311 ± 3	<0.0001	191 ± 3	267 ± 3	362 ± 3	<0.0001
Thiamin (mg)	0.89± 0.01	0.96 ± 0.01	1.00 ± 0.01	<0.0001	1.16 ± 0.02	1.22 ± 0.02	1.24 ± 0.02	0.007	0.90 ± 0.01	0.99 ± 0.01	1.05 ± 0.01	<0.0001	0.90 ± 0.01	0.98 ± 0.01	1.05 ± 0.01	<0.0001
Riboflavin (mg)	1.15 ± 0.01	1.23 ± 0.01	1.29 ±0.01	<0.0001	1.27 ± 0.02	1.42 ± 0.02	1.52 ± 0.02	<0.0001	0.99 ± 0.01	1.14 ± 0.01	1.29 ± 0.01	<0.0001	1.12 ± 0.01	1.27 ± 0.01	1.44 ± 0.01	<0.0001
Niacin (mg)	12.4 ± 0.2	13.5 ± 0.2	14.1 ± 0.2	<0.0001	16.4 ± 0.3	17.4 ± 0.3	18.7 ± 0.3	<0.0001	15.2 ± 0.1	17.4 ± 0.1	19.2 ± 0.1	<0.0001	16.1 ± 0.1	18.1 ± 0.1	19.7 ± 0.1	<0.0001
Vitamin B-6 (mg)	1.0 ± 0.01	1.1 ± 0.01	1.2 ± 0.01	<0.0001	1.2 ± 0.02	1.4 ± 0.02	1.6 ± 0.02	<0.0001	1.0 ± 0.01	1.2 ± 0.1	1.5 ± 0.1	<0.0001	1.2 ± 0.01	1.4 ± 0.01	1.6 ± 0.01	<0.0001
Vitamin B-12 (μg)	4.6 ± 0.2	5.1 ± 0.2	5.7 ± 0.2	0.0003	4.9 ± 0.3	6.7 ± 0.3	7.3 ± 0.3	<0.0001	4.4 ± 0.1	5.8 ± 0.1	6.8 ± 0.1	<0.0001	6.2 ± 0.1	7.6 ± 0.1	8.3 ± 0.1	<0.0001
Folate (μg)	225 ± 3.5	268 ± 3.5	296 ± 3.5	<0.0001	259 ± 5.3	318 ± 5.3	380 ± 5.3	<0.0001	225 ± 2.7	307 ± 2.7	399 ± 2.7	<0.0001	301 ± 2.3	396 ± 2.3	481 ± 2.3	<0.0001
Pantothenic acid (mg)	5.9 ± 0.1	6.4 ± 0.1	6.7 ± 0.1	<0.0001	6.8 ± 0.1	7.5 ± 0.1	8.1 ± 0.1	<0.0001	5.2 ± 0.03	6.0 ± 0.03	6.7 ± 0.03	<0.0001	5.5 ± 0.02	6.3 ± 0.02	7.0 ± 0.02	<0.0001
Vitamin C (mg)	76.5 ± 2.3	90.9 ± 2.3	104 ± 2.3	<0.0001	91.1 ± 3.3	107 ± 3.3	132 ± 3.3	<0.0001	67.0 ± 1.3	99.4 ± 1.3	135 ± 1.3	<0.0001	107 ± 1.2	153 ± 1.2	188 ± 1.2	<0.0001
**Minerals**																
Sodium(gNaClequivalent)	8.4 ± 0.1	8.4 ± 0.1	7.6 ± 0.1	<0.0001	10.4 ± 0.2	10.5 ± 0.2	9.8 ± 0.2	0.003	10.4 ± 0.1	10.3 ± 0.1	9.6 ± 0.1	<0.0001	11.7 ± 0.1	11.4 ± 0.1	10.0 ± 0.1	<0.0001
Potassium (mg)	2133 ± 21	2407 ± 21	2599 ± 21	<0.0001	2330 ± 29	2746 ± 29	3124 ± 29	<0.0001	1967 ± 13	2397 ± 13	2886 ± 13	<0.0001	2371 ± 10	2905 ± 11	3370 ± 11	<0.0001
Calcium (mg)	590 ± 9	653 ± 9	693 ± 9	<0.0001	527 ± 12	655 ± 12	719 ± 12	<0.0001	372 ± 4	440 ± 4	529 ± 4	<0.0001	449 ± 3	535 ± 3	650 ± 3	<0.0001
Magnesium (mg)	203 ± 2	226 ± 2	248 ± 2	<0.0001	226 ± 3	266 ± 3	302 ± 3	<0.0001	206 ± 1	245 ± 1	284 ± 1	<0.0001	246 ± 1	287 ± 1	325 ± 1	<0.0001
Phosphorus (mg)	1003 ± 8	1072 ± 8	1117 ± 8	<0.0001	1123 ± 11	1257 ± 11	1325 ± 11	<0.0001	887 ± 4	1007 ± 4	1102 ± 4	<0.0001	970 ± 3	1078 ± 3	1177 ± 3	<0.0001
Iron (mg)	5.8 ± 0.07	6.5 ± 0.1	7.2 ± 0.1	<0.0001	7.0 ± 0.1	8.0 ± 0.1	9.0 ± 0.1	<0.0001	6.2 ± 0.1	7.4 ± 0.1	8.7 ± 0.1	<0.0001	7.5 ± 0.04	8.8 ± 0.04	9.9 ± 0.04	<0.0001
Zinc (mg)	7.7 ± 0.1	8.1 ± 0.1	8.4 ± 0.1	<0.0001	9.8 ± 0.1	10.6 ± 0.1	10.8 ± 0.1	<0.0001	7.8 ± 0.04	8.5 ± 0.04	9.0 ± 0.04	<0.0001	7.9 ± 0.03	8.5 ± 0.03	8.9 ± 0.03	<0.0001
Copper (mg)	0.9 ± 0.01	1.0 ± 0.01	1.1 ± 0.01	<0.0001	1.2 ± 0.01	1.3 ± 0.01	1.4 ± 0.01	<0.0001	1.0 ± 0.01	1.1 ± 0.01	1.3 ± 0.01	<0.0001	1.1 ± 0.01	1.3 ± 0.01	1.4 ± 0.01	<0.0001
Manganese (mg)	2.2 ± 0.03	2.5 ± 0.03	2.8 ± 0.03	<0.0001	3.0 ± 0.1	3.3 ± 0.1	3.6 ± 0.1	<0.0001	2.8 ± 0.03	3.3 ± 0.03	3.8 ± 0.03	<0.0001	3.6 ± 0.02	4.1 ± 0.02	4.5 ± 0.02	<0.0001

RAE, retinol activity equivalent. [1] Values are means ± SEs unless otherwise indicated. Adjustment was made for age, sex, weight status, occupation (for younger and older adults only), and total energy intake. NRF9.3 score was calculated based on daily intake of nine nutrients to encourage (i.e., protein, dietary fiber, vitamins A, C, and D, calcium, iron, potassium, and magnesium) and three nutrients to limit (i.e., added sugar, saturated fat, and sodium). A higher score indicates a higher diet quality. [2] Calculated by using general linear models.

Table S5. Daily intakes of food groups (in grams) according to tertile (T) category of the Nutrient-Rich Food Index 9.3 (NRF9.3) score as a measure of overall diet quality [1].

	Children (aged 6–11 years)			p for	Adolescents (aged 12–17 years)			p for	Younger adults (aged 18–49 years)			p for	Older adults (aged ≥ 50 years)			p for
	T1	T2	T3	Trend [2]	T1	T2	T3	Trend [2]	T1	T2	T3	Trend [2]	T1	T2	T3	Trend [2]

n	**481**	**482**	**481**		**378**	**378**	**378**		**2177**	**2177**	**2177**		**4447**	**4448**	**4448**	
Rice	258 ± 5.2	289 ± 5.2	325 ± 5.2	<0.0001	411 ± 9.0	432 ± 8.9	469 ± 8.9	<0.0001	323 ± 3.4	354 ± 3.4	379 ± 3.4	<0.0001	335 ± 2.3	340 ± 2.3	337 ± 2.3	0.56
Bread	41.3 ± 2.0	38.3 ± 2.0	28.3 ± 2.0	<0.0001	37.6 ± 2.6	43.5 ± 2.6	33.6 ± 2.6	0.27	33.5 ± 1.0	32.2 ± 1.0	27.2 ± 1.0	<0.0001	31.7 ± 0.6	30.7 ± 0.6	29.8 ± 0.6	0.03
Noodles	39.7 ± 2.7	28.5 ± 2.7	18.5 ± 2.7	<0.0001	68.4 ± 5.3	49.7 ± 5.2	29.3 ± 5.2	<0.0001	103 ± 2.4	72.6 ± 2.4	45.8 ± 2.4	<0.0001	83.5 ± 1.5	60.7 ± 1.5	43 ± 1.5	<0.0001
Other grains	19.9 ± 1.6	25.5 ± 1.6	24.2 ± 1.6	0.054	23.2 ± 1.9	24.7 ± 1.9	22.3 ± 1.9	0.73	17.2 ± 0.8	17.5 ± 0.8	15.7 ± 0.8	0.18	15.1 ± 0.5	14.5 ± 0.5	14.6 ± 0.5	0.43
Potatoes	47.0 ± 2.2	56.7 ± 2.2	66.6 ± 2.2	<0.0001	45.0 ± 3.4	69.9 ± 3.3	80.0 ± 3.4	<0.0001	37.2 ± 1.3	50.7 ± 1.3	61.6 ± 1.3	<0.0001	48.7 ± 1.0	63.3 ± 1.0	67.4 ± 1.0	<0.0001
Sugar	7.6 ± 0.3	6.8 ± 0.3	5.6 ± 0.3	<0.0001	8.2 ± 0.4	7.2 ± 0.4	6.8 ± 0.4	0.03	7.5 ± 0.2	6.4 ± 0.2	6.3 ± 0.2	<0.0001	10.8 ± 0.2	8.8 ± 0.2	7.4 ± 0.2	<0.0001
Pulses	34.9 ± 2.4	45.3 ± 2.4	60.0 ± 2.4	<0.0001	31.5 ± 2.9	50.5 ± 2.8	66.7 ± 2.8	<0.0001	35.3 ± 1.6	54.3 ± 1.6	70.5 ± 1.6	<0.0001	56.1 ± 1.2	72.1 ± 1.2	87.2 ± 1.2	<0.0001
Nuts	1.1 ± 0.2	2.0 ± 0.2	2.1 ± 0.2	0.002	1.2 ± 0.3	1.7 ± 0.3	2.4 ± 0.3	0.003	1.1 ± 0.1	1.6 ± 0.1	2.4 ± 0.1	<0.0001	1.8 ± 0.2	2.6 ± 0.2	4.1 ± 0.2	<0.0001
Vegetables	188 ± 4.1	241 ± 4.1	252 ± 4.1	<0.0001	222 ± 5.9	279 ± 5.8	337 ± 5.8	<0.0001	188 ± 2.9	267 ± 2.9	354 ± 2.9	<0.0001	245 ± 2.4	338 ± 2.4	404 ± 2.4	<0.0001
Fruits	47.8 ± 3.5	63.3 ± 3.5	90.6 ± 3.5	<0.0001	37.0 ± 4.7	61.2 ± 4.7	91.8 ± 4.7	<0.0001	30.1 ± 1.7	50.7 ± 1.7	75.7 ± 1.7	<0.0001	91.7 ± 2.0	143 ± 2.0	184 ± 2.0	<0.0001
Fish	38.4 ± 2.0	47.6 ± 2	54.7 ± 2.0	<0.0001	41.0 ± 3.0	62.3 ± 2.9	67.7 ± 2.9	<0.0001	44.3 ± 1.4	62.4 ± 1.4	76.6 ± 1.4	<0.0001	72.8 ± 1.1	91.1 ± 1.1	102 ± 1.1	<0.0001
Meat	92.3 ± 2.2	84.3 ± 2.2	76.4 ± 2.2	<0.0001	144 ± 3.8	130 ± 3.8	117 ± 3.8	<0.0001	110 ± 1.4	106 ± 1.4	95 ± 1.4	<0.0001	78 ± 0.9	70 ± 0.9	61 ± 0.9	<0.0001
Eggs	26.0 ± 1.3	27.7 ± 1.3	29.5 ± 1.3	0.053	38.9 ± 1.9	40.3 ± 1.9	44.3 ± 1.9	0.04	29.4 ± 0.7	36.5 ± 0.7	40.3 ± 0.7	<0.0001	32.5 ± 0.5	34.8 ± 0.5	35.5 ± 0.5	<0.0001
Dairy products	274 ± 6.7	309 ± 6.7	315 ± 6.7	<0.0001	182 ± 9.1	257 ± 9.0	271 ± 9.1	<0.0001	67.1 ± 2.6	81.2 ± 2.6	98.7 ± 2.6	<0.0001	76.1 ± 1.8	96.0 ± 1.8	133 ± 1.8	<0.0001
Fats and oils	9.3 ± 0.3	8.8 ± 0.3	8.2 ± 0.3	0.0115	14.0 ± 0.5	12.5 ± 0.5	11.4 ± 0.5	<0.0001	12.3 ± 0.2	12 ± 0.2	10.7 ± 0.2	<0.0001	9.6 ± 0.1	9.1 ± 0.1	7.7 ± 0.1	<0.0001
Confectioneries	53.7 ± 2.2	37.4 ± 2.2	33.3 ± 2.2	<0.0001	62.2 ± 3.1	38 ± 3.1	33.3 ± 3.1	<0.0001	40.7 ± 1.1	28.1 ± 1.1	20.7 ± 1.1	<0.0001	40.3 ± 0.7	26.9 ± 0.7	19.2 ± 0.7	<0.0001
Fruit juice	21.2 ± 2.3	11.6 ± 2.3	5.5 ± 2.3	<0.0001	30.7 ± 3.8	15.8 ± 3.7	13.0 ± 3.7	0.001	12.3 ± 1.1	6.4 ± 1.0	5.7 ± 1	<0.0001	6.1 ± 0.5	3.7 ± 0.5	5.1 ± 0.5	0.14
Vegetable juice	4.4 ± 1.5	8.5 ± 1.5	7.8 ± 1.5	0.11	11.1 ± 3.0	7.4 ± 2.9	15.7 ± 3.0	0.27	4.5 ± 1.1	10.0 ± 1.1	14.3 ± 1.1	<0.0001	6.2 ± 0.7	9.8 ± 0.7	12.4 ± 0.7	<0.0001
Soft drinks	104 ± 4.9	28.3 ± 4.9	17.8 ± 4.9	<0.0001	133 ± 8.5	36.7 ± 8.5	18.0 ± 8.5	<0.0001	118 ± 3.4	52.8 ± 3.4	27.6 ± 3.4	<0.0001	57.9 ± 1.5	30.9 ± 1.5	18.9 ± 1.5	<0.0001
Tea and coffee	201 ± 11.1	218 ± 11.1	213 ± 11.1	0.44	286 ± 16.7	287 ± 16.6	308 ± 16.6	0.35	447 ± 8.5	475 ± 8.4	513 ± 8.4	<0.0001	489 ± 6	546 ± 6	566 ± 6	<0.0001
Seasonings	66.0 ± 2.6	59.4 ± 2.6	58.7 ± 2.6	0.048	71.0 ± 3.0	76.8 ± 3.0	72.9 ± 3.0	0.67	97.1 ± 1.9	93.3 ± 1.9	79.7 ± 1.9	<0.0001	94.9 ± 1.2	89.4 ± 1.2	79.5 ± 1.2	<0.0001

[1] Values are means ± SEs unless otherwise indicated. Adjustment was made for age, sex, weight status, occupation (for younger and older adults only), and total energy intake. NRF9.3 score was calculated based on daily intake of nine nutrients to encourage (i.e., protein, dietary fiber, vitamins A, C, and D, calcium, iron, potassium, and magnesium) and three nutrients to limit (i.e., added sugar, saturated fat, and sodium). A higher score indicates a higher diet quality. [2] Calculated by using general linear models.

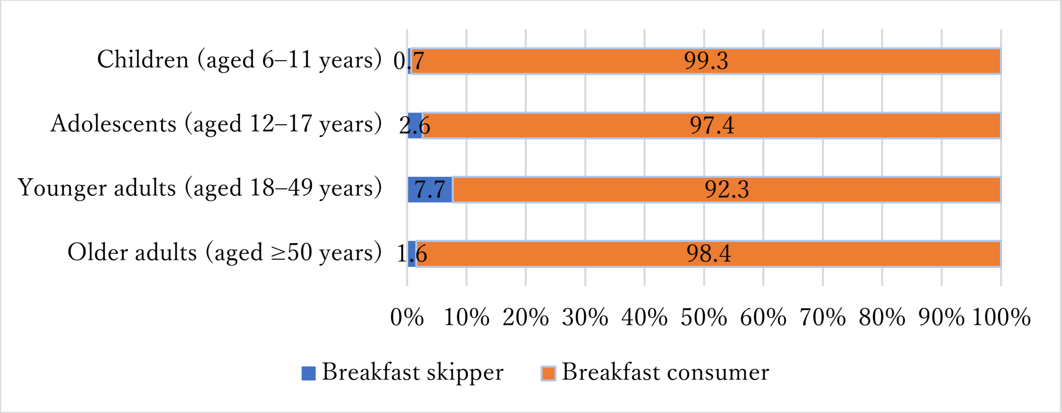

Figure S1. Proportion of breakfast skippers and consumers. Total n = 1444 for children, 1144 for adolescents, 6531 for younger adults, and 13343 for older adults.

Table S6. Daily intakes of breakfast skippers and consumers: Nutrient-Rich Food Index 9.3 (NRF9.3) score, energy, and macronutrients [1].

	**Children (aged 6–11 years)**			**Adolescents (aged 12–17 years)**			**Younger adults (aged 18–49 years)**			**Older adults (aged ≥ 50 years)**		
	**Breakfast Skipper**	**Breakfast Consumer**	**P[2]**	**Breakfast Skipper**	**Breakfast Consumer**	**p[2]**	**Breakfast Skipper**	**Breakfast Consumer**	**p[2]**	**Breakfast Skipper**	**Breakfast Consumer**	**p[2]**
n	**10**	**1434**		**30**	**1104**		**503**	**6028**		**208**	**13135**	
NRF9.3 score	558 ± 33	684 ± 3	0.0001	543 ± 22	637 ± 4	<0.0001	527 ± 6	599 ± 2	<0.0001	601 ± 8	669 ± 1	<0.0001
Energy (kcal)	1686 ± 117	1800 ± 10	0.33	1814 ± 108	2305 ± 18	<0.0001	1621 ± 24	1978 ± 7	<0.0001	1547 ± 34	1937 ± 4	<0.0001
**Macronutrients (g)**												
Protein	55.9 ± 3.2	66.1 ± 0.3	0.002	75.0 ± 2.6	82.7 ± 0.4	0.004	66.6 ± 0.7	69.8 ± 0.2	<0.0001	70.6 ± 1.0	72.3 ± 0.1	0.09
Fat	65.0 ± 3.7	59.6 ± 0.3	0.15	76.1 ± 3.2	74.9 ± 0.5	0.72	62.8 ± 0.7	60.1 ± 0.2	0.0006	58.0 ± 1.1	52.8 ± 0.1	<0.0001
SFA	22.5 ± 1.6	20.1 ± 0.1	0.14	23.0 ± 1.2	23.5 ± 0.2	0.70	18.2 ± 0.3	17.1 ± 0.1	0.0002	15.9 ± 0.4	14.4 ± 0.1	<0.0001
MUFA	23.5 ± 1.6	20.5 ± 0.1	0.08	28.2 ± 1.4	27.2 ± 0.2	0.47	24.1 ± 0.3	22.3 ± 0.1	<0.0001	21.6 ± 0.5	18.7 ± 0.1	<0.0001
n-6 PUFA	10.4 ± 1.0	9.3 ± 0.1	0.25	12.6 ± 0.8	12.2 ± 0.1	0.58	10.9 ± 0.2	10.8 ± 0.1	0.51	10.0 ± 0.3	9.7 ± 0.03	0.38
n-3 PUFA	1.7 ± 0.3	1.8 ± 0.03	0.70	2.5 ± 0.2	2.3 ± 0.04	0.53	2.2 ± 0.1	2.2 ± 0.01	0.70	2.6 ± 0.1	2.6 ± 0.01	0.50
Carbohydrate	241.0 ± 9.4	243.7 ± 0.8	0.78	315.9 ± 8.0	312.1 ± 1.3	0.64	252.6 ± 2.0	263.5 ± 0.6	<0.0001	258.2 ± 3.1	272.9 ± 0.4	<0.0001
Added sugar	38.7 ± 6.4	26.4 ± 0.5	0.06	36.4 ± 4.4	30.2 ± 0.7	0.17	29.7 ± 1.0	25.0 ± 0.3	<0.0001	28.7 ± 1.3	25.1 ± 0.2	0.008
Dietary fiber	10.1 ± 1.0	12.0 ± 0.1	0.06	11.8 ± 0.7	14.2 ± 0.1	0.002	11.6 ± 0.2	13.1 ± 0.1	<0.0001	15.1 ± 0.4	16.4 ± 0.1	0.002
**Macronutrient balance (% energy)**												
Protein	12.6 ± 0.7	14.8 ± 0.1	0.002	13.7 ± 0.5	14.6 ± 0.1	0.06	14.2 ± 0.1	14.4 ± 0.04	0.11	15.1 ± 0.2	15.1 ± 0.03	0.79
Fat	31.5 ± 1.8	29.6 ± 0.2	0.30	28.9 ± 1.2	29.3 ± 0.2	0.75	28.3 ± 0.3	27.6 ± 0.1	0.04	25.6 ± 0.5	24.3 ± 0.1	0.008
SFA	11.1 ± 0.8	10.0 ± 0.1	0.16	8.7 ± 0.4	9.2 ± 0.1	0.27	8.1 ± 0.1	7.8 ± 0.04	0.09	6.8 ± 0.2	6.6 ± 0.02	0.34
MUFA	11.1 ± 0.8	10.2 ± 0.1	0.23	10.7 ± 0.5	10.6 ± 0.1	0.81	10.8 ± 0.2	10.2 ± 0.04	0.0003	9.4 ± 0.2	8.6 ± 0.03	<0.0001
PUFA	5.9 ± 0.5	5.6 ± 0.04	0.56	5.7 ± 0.3	5.7 ± 0.1	0.98	6.1 ± 0.1	6.0 ± 0.03	0.19	5.8 ± 0.1	5.7 ± 0.02	0.35
Carbohydrate	54.4 ± 2.0	54.4 ± 0.2	0.98	55.3 ± 1.3	54.5 ± 0.2	0.51	52.8 ± 0.4	54.4 ± 0.1	0.0002	55.1 ± 0.6	57.1 ± 0.1	0.0009
Added sugar	8.2 ± 1.3	5.8 ± 0.1	0.06	5.3 ± 0.7	5.2 ± 0.1	0.87	5.5 ± 0.2	5.1 ± 0.1	0.06	5.5 ± 0.3	5.2 ± 0.03	0.24

[1] Values are means ± SEs unless otherwise indicated. Adjustment was made for age, sex, weight status, occupation (for younger and older adults only), and total energy intake (except for the analysis on energy intake itself). NRF9.3 score was calculated based on daily intake of nine nutrients to encourage (i.e., protein, dietary fiber, vitamins A, C, and D, calcium, iron, potassium, and magnesium) and three nutrients to limit (i.e., added sugar, saturated fat, and sodium). A higher score indicates a higher diet quality. [2] Calculated by using general linear models.

Table S7. Daily intakes of breakfast skippers and consumers: vitamins and minerals [1].

	Children (aged 6–11 years)			Adolescents (aged 12–17 years)			Younger adults (aged 18–49 years)			Older adults (aged ≥ 50 years)		
	**Breakfast Skipper**	**Breakfast Consumer**	**p[2]**	**Breakfast Skipper**	**Breakfast Consumer**	**p[2]**	**Breakfast Sskipper**	**Breakfast Consumer**	**p[2]**	**Breakfast Skipper**	**Breakfast Consumer**	**p[2]**
n	**10**	**1434**		**30**	**1104**		**503**	**6028**		**208**	**13135**	
**Vitamins**												
Vitamin A (μg RAE)	309.3 ± 99.5	527.1 ± 8.3	0.03	445.0 ± 108.7	619.7 ± 17.7	0.11	470.1 ± 34.1	508.3 ± 9.6	0.28	599.3 ± 62.5	595.4 ± 7.8	0.95
Vitamin D (μg)	6.9 ± 2.0	6.0 ± 0.2	0.65	6.5 ± 1.6	7.3 ± 0.3	0.60	5.6 ± 0.4	6.6 ± 0.1	0.02	8.4 ± 0.7	9.3 ± 0.1	0.19
Vitamin E (mg)	5.3 ± 0.6	6.0 ± 0.1	0.27	7.0 ± 0.5	7.5 ± 0.1	0.33	6.4 ± 0.1	6.7 ± 0.04	0.01	7.3 ± 0.2	7.3 ± 0.03	0.99
Vitamin K (μg)	90.4 ± 37.1	180.1 ± 3.1	0.02	148.5 ± 26.5	226.4 ± 4.3	0.004	190.3 ± 7.3	228.1 ± 2.1	<0.0001	222.4 ± 13	274.1 ± 1.6	<0.0001
Thiamin (mg)	1.01 ± 0.1	0.95 ± 0.01	0.46	1.13 ± 0.1	1.21 ± 0.01	0.32	0.97 ± 0.02	0.98 ± 0.01	0.63	1.00 ± 0.02	0.97 ± 0.003	0.41
Riboflavin (mg)	0.97 ± 0.1	1.23 ± 0.01	0.003	1.27 ± 0.1	1.41 ± 0.01	0.08	1.06 ± 0.02	1.15 ± 0.01	<0.0001	1.27 ± 0.03	1.28 ± 0.004	0.76
Niacin (mg)	12.1 ± 1.3	13.3 ± 0.1	0.38	15.9 ± 1.15	17.5 ± 0.2	0.17	16.99 ± 0.3	17.3 ± 0.1	0.30	18.5 ± 0.5	17.9 ± 0.1	0.19
Vitamin B-6 (mg)	0.94 ± 0.09	1.13 ± 0.01	0.03	1.16 ± 0.07	1.40 ± 0.01	0.0005	1.16 ± 0.02	1.23 ± 0.01	<0.0001	1.40 ± 0.03	1.40 ± 0.004	0.22
Vitamin B-12 (μg)	5.7 ± 1.4	5.2 ± 0.1	0.69	5.9 ± 1.1	6.3 ± 0.2	0.71	5.5 ± 0.3	5.7 ± 0.1	0.60	7.5 ± 0.5	7.6 ± 0.1	0.91
Folate (μg)	172.0 ± 26.0	263.6 ± 2.2	0.0005	231.2 ± 20.9	321.4 ± 3.4	<0.0001	270.0 ± 6.6	313.6 ± 1.9	<0.0001	350.2 ± 11.8	393.4 ± 1.5	0.0003
Pantothenic acid (mg)	5.4 ± 0.4	6.3 ± 0.03	0.02	6.5 ± 0.3	7.5 ± 0.1	0.0005	5.6 ± 0.1	6.0 ± 0.02	<0.0001	6.1 ± 0.1	6.3 ± 0.01	0.11
Vitamin C (mg)	51.3 ± 16.0	90.7 ± 1.3	0.01	89.9 ± 12.1	110.7 ± 2.0	0.09	86.7 ± 3.0	101.5 ± 0.9	<0.0001	134.1 ± 6	149.4 ± 0.8	0.01
**Minerals**												
Sodium (g NaCl equivalent)	8.1 ± 0.7	8.1 ± 0.1	0.96	9.8 ± 0.6	10.3 ± 0.1	0.42	9.5 ± 0.2	10.1 ± 0.04	0.0002	10.5 ± 0.3	11.1 ± 0.03	0.02
Potassium (mg)	2100 ± 156	2382 ± 13	0.07	2345 ± 118	2744 ± 19	0.0009	2215 ± 33	2434 ± 9	<0.0001	2743 ± 58	2884 ± 7	0.02
Calcium (mg)	519.7 ± 60.6	646.1 ± 5.1	0.04	535.3 ± 45.9	636.4 ± 7.5	0.03	393.0 ± 9.7	451.6 ± 2.7	<0.0001	506.6 ± 16.3	545.2 ± 2.0	0.02
Magnesium (mg)	194.2 ± 15.4	226.0 ± 1.3	0.04	229.4 ± 11.1	265.3 ± 1.8	0.002	225.7 ± 3.1	246.3 ± 0.9	<0.0001	269.9 ± 5.3	286.2 ± 0.7	0.002
Phosphorus (mg)	887.0 ± 55.7	1064.9 ± 4.6	0.002	1081.2 ± 42.1	1239.1 ± 6.8	0.0002	941.3 ± 10.1	1003.5 ± 2.9	<0.0001	1040.7 ± 16.2	1075.5 ± 2	0.03
Iron (mg)	5.8 ± 0.5	6.5 ± 0.05	0.18	6.9 ± 0.4	8.1 ± 0.1	0.004	6.9 ± 0.1	7.5 ± 0.03	<0.0001	8.2 ± 0.2	8.7 ± 0.02	0.009
Zinc (mg)	7.1 ± 0.4	8.1 ± 0.04	0.02	9.3 ± 0.4	10.4 ± 0.1	0.004	8.3 ± 0.1	8.4 ± 0.03	0.17	8.3 ± 0.1	8.4 ± 0.02	0.34
Copper (mg)	0.9 ± 0.1	1.0 ± 0.01	0.08	1.2 ± 0.1	1.3 ± 0.01	0.10	1.1 ± 0.01	1.2 ± 0.004	0.0005	1.2 ± 0.02	1.3 ± 0.003	0.17
Manganese (mg)	2.2 ± 0.2	2.5 ± 0.02	0.31	3.1 ± 0.2	3.3 ± 0.03	0.18	3.1 ± 0.1	3.3 ± 0.02	0.0003	3.6 ± 0.1	4.1 ± 0.01	<0.0001

RAE, retinol activity equivalent. [1] Values are means ± SEs unless otherwise indicated. Adjustment was made for age, sex, weight status, occupation (for younger and older adults only), and total energy intake. [2] Calculated by using general linear models.

Table S8. Daily intakes of breakfast skippers and consumers: food groups (in grams)[1].

	Children (aged 6–11 years)			Adolescents (aged 12–17 years)			Younger adults (aged 18–49 years)			Older adults (aged ≥ 50 years)		
	**Breakfast Skipper**	**Breakfast Consumer**	**p[2]**	**Breakfast Skipper**	**Breakfast Consumer**	**p[2]**	**Breakfast Skipper**	**Breakfast Consumer**	**p[2]**	**Breakfast Skipper**	**Breakfast Consumer**	**p[2]**
n	**10**	**1434**		**30**	**1104**		**503**	**6028**		**208**	**13135**	
Rice	269.3 ± 36.9	290.9 ± 3.1	0.56	444.7 ± 32.2	437 ± 5.2	0.81	336.2 ± 7.2	353.2 ± 2.0	0.02	311.9 ± 10.6	337.8 ± 1.3	0.02
Bread	20.3 ± 14.1	36.1 ± 1.2	0.27	17.8 ± 9.2	38.8 ± 1.5	0.02	14.9 ± 2.0	32.3 ± 0.6	<0.0001	16.6 ± 2.9	31.0 ± 0.4	<0.0001
Noodles	44.1 ± 19.1	28.8 ± 1.6	0.43	79.0 ± 18.9	48.3 ± 3.1	0.11	82.7 ± 5.2	73.2 ± 1.5	0.08	65.8 ± 7.1	62.4 ± 0.9	0.63
Other grains	11.1 ± 10.9	23.3 ± 0.9	0.27	21.9 ± 6.7	23.4 ± 1.1	0.82	17.6 ± 1.6	16.7 ± 0.5	0.62	19.5 ± 2.4	14.7 ± 0.3	0.04
Potatoes	28.4 ± 15.1	57.0 ± 1.3	0.06	67.8 ± 12.3	64.9 ± 2.0	0.81	48.8 ± 2.7	49.9 ± 0.8	0.70	65.3 ± 4.8	59.8 ± 0.6	0.26
Sugar	5.0 ± 2.3	6.7 ± 0.2	0.47	4.7 ± 1.6	7.5 ± 0.3	0.09	5.9 ± 0.4	6.8 ± 0.1	0.02	8.0 ± 0.7	9.0 ± 0.1	0.16
Pulses	29.7 ± 16.7	46.9 ± 1.4	0.31	42.8 ± 10.5	49.8 ± 1.7	0.52	43.3 ± 3.4	54.2 ± 1.0	0.002	68.5 ± 5.5	71.8 ± 0.7	0.55
Nuts	4.1 ± 1.6	1.7 ± 0.1	0.13	0.8 ± 1.1	1.8 ± 0.2	0.34	1.9 ± 0.3	1.6 ± 0.1	0.39	2.9 ± 0.7	2.8 ± 0.1	0.93
Vegetables	182.0 ± 29.4	227.2 ± 2.5	0.13	196.6 ± 22.5	281.3 ± 3.7	0.0002	245.3 ± 6.9	271.9 ± 2.0	0.0002	295.5 ± 11.9	329.8 ± 1.5	0.004
Fruits	12.8 ± 25.0	67.6 ± 2.1	0.03	23.8 ± 17.3	26.4 ± 2.8	0.02	39.4 ± 3.7	53.2 ± 1.1	0.0004	125.0 ± 9.6	139.7 ± 1.2	0.13
Fish	31.3 ± 13.9	47.0 ± 1.2	0.26	58.8 ± 10.7	56.9 ± 1.7	0.86	60.0 ± 3.0	61.2 ± 0.8	0.71	88.2 ± 5.1	88.6 ± 0.6	0.94
Meat	95.1 ± 15.3	84.2 ± 1.3	0.48	130.9 ± 13.8	130.2 ± 2.2	0.96	115.3 ± 3.0	102.5 ± 0.9	<0.0001	82.5 ± 4.0	69.3 ± 0.5	0.001
Eggs	4.7 ± 8.8	27.9 ± 0.7	0.009	22.6 ± 6.7	41.7 ± 1.1	0.005	28.8 ± 1.5	35.9 ± 0.4	<0.0001	31.6 ± 2.3	34.3 ± 0.3	0.24
Dairy products	257.1 ± 46.6	299.5 ± 3.9	0.36	167.2 ± 33.2	238.4 ± 5.4	0.03	51.3 ± 5.5	84.9 ± 1.6	<0.0001	80.6 ± 8.5	101.9 ± 1.1	0.01
Fats and oils	5.0 ± 2.1	8.8 ± 0.2	0.07	11.9 ± 1.7	12.6 ± 0.3	0.68	12.5 ± 0.4	11.6 ± 0.1	0.04	9.4 ± 0.6	8.8 ± 0.1	0.28
Confectioneries	78.5 ± 15.3	41.2 ± 1.3	0.02	67.3 ± 11.2	43.9 ± 1.8	0.04	36.5 ± 2.3	29.3 ± 0.7	0.003	38.3 ± 3.3	28.6 ± 0.4	0.003
Fruit juice	17.0 ± 16.1	12.7 ± 1.3	0.79	4.2 ± 13.4	20.2 ± 2.2	0.24	8.1 ± 2.2	8.1 ± 0.6	0.98	4.4 ± 2.2	5.0 ± 0.3	0.80
Vegetable juice	0.6 ± 10.6	6.9 ± 0.9	0.48	50.4 ± 10.5	10.4 ± 1.7	0.0002	4.3 ± 2.3	10.0 ± 0.6	0.02	5.4 ± 3.2	9.5 ± 0.4	0.20
Soft drinks	106.9 ± 36.0	49.6 ± 3.0	0.11	142.6 ± 31.7	60.3 ± 5.2	0.01	84.5 ± 7.4	64.6 ± 2.1	0.01	52.5 ± 7.1	35.7 ± 0.9	0.02
Tea and coffee	75.0 ± 76.5	211.8 ± 6.4	0.07	232.2 ± 59.5	295.4 ± 9.7	0.29	389.6 ± 18.0	486.0 ± 5.7	<0.0001	385.6 ± 27.8	535.6 ± 3.5	<0.0001
Seasonings	73.9 ± 18	61.3 ± 1.5	0.48	56.2 ± 10.7	74.1 ± 1.7	0.10	82.5 ± 4.1	90.6 ± 1.2	0.06	82.6 ± 5.8	88.0 ± 0.7	0.36

[1] Values are means ± SEs unless otherwise indicated. Adjustment was made for age, sex, weight status, occupation (for younger and older adults only), and total energy intake.

[2] Calculated by using general linear models.

Table S9. Intakes of food groups (in grams) and percentage of consumers at breakfast among breakfast consumers[1].

	Children (aged 6–11 years)	Adolescents (aged 12–17 years)	Younger adults (aged 18–49 years)	Older adults (aged ≥ 50 years)
n	**1434**	**1104**	**6028**	**13135**
Rice	64.4 ± 66.2 (57%)	93.0 ± 97.6 (58%)	77.3 ± 93.4 (47%)	93.6 ± 89.6 (60%)
Bread	18.9 ± 30.1 (34%)	22.1 ± 35.5 (33%)	24.0 ± 37.1 (34%)	21.6 ± 34.6 (32%)
Noodles	1.7 ± 14.7 (2%)	3.9 ± 27.0 (3%)	2.9 ± 23.4 (2%)	1.5 ± 16.0 (1%)
Other grains	3.1 ± 13.7 (11%)	3.4 ± 16.0 (10%)	2.3 ± 12.8 (7%)	2.0 ± 12.5 (7%)
Potatoes	5.0 ± 15.2 (18%)	4.6 ± 15.9 (14%)	5.4 ± 18.7 (14%)	9.7 ± 25.9 (21%)
Sugar	1.6 ± 4.1 (28%)	2.0 ± 5.1 (31%)	2.0 ± 5.0 (30%)	2.9 ± 6.2 (40%)
Pulses	9.5 ± 19.7 (29%)	12.9 ± 26.4 (33%)	15.8 ± 36.3 (31%)	25 ± 41.8 (48%)
Nuts	0.2 ± 1.6 (5%)	0.3 ± 2.8 (5%)	0.3 ± 2.5 (5%)	0.5 ± 2.8 (8%)
Vegetables	24.2 ± 36.1 (60%)	32.0 ± 43.2 (63%)	36.3 ± 56.0 (56%)	71.6 ± 78.3 (76%)
Fruits	16.8 ± 37.5 (26%)	20.3 ± 46.6 (26%)	19.4 ± 44.9 (24%)	41.1 ± 66.3 (43%)
Fish	3.8 ± 12.3 (18%)	5.6 ± 17.0 (20%)	6.6 ± 20.7 (20%)	11.9 ± 26.0 (34%)
Meat	12.5 ± 20.6 (43%)	15.2 ± 23.1 (46%)	9.7 ± 21.2 (30%)	6.2 ± 16.4 (22%)
Eggs	14.0 ± 20.4 (42%)	15.2 ± 22.2 (42%)	12.1 ± 21.0 (31%)	14.3 ± 22.7 (34%)
Dairy products	59.5 ± 86.4 (45%)	64.8 ± 93.4 (47%)	44.6 ± 80.2 (41%)	55.7 ± 88.2 (45%)
Fats and oils	1.8 ± 3.1 (50%)	2.3 ± 3.5 (53%)	2.2 ± 4.0 (43%)	1.9 ± 3.6 (40%)
Confectioneries	5.1 ± 21.5 (9%)	5.5 ± 24.0 (8%)	5.4 ± 24.6 (7%)	2.7 ± 16.6 (4%)
Fruit juice	5.0 ± 28.8 (3%)	6.1 ± 34.3 (3%)	2.7 ± 23.0 (2%)	1.9 ± 18.1 (1%)
Vegetable juice	4.6 ± 26.5 (3%)	5.7 ± 33.1 (3%)	4.4 ± 28.9 (3%)	4.7 ± 29.3 (3%)
Soft drinks	11.3 ± 39.8 (14%)	7.7 ± 34.1 (10%)	12.7 ± 52.3 (9%)	7.1 ± 33.3 (7%)
Tea and coffee	41.0 ± 69.6 (31%)	59.2 ± 89.2 (37%)	119.3 ± 130.0 (60%)	145.3 ± 140.2 (68%)
Seasonings	9.7 ± 20.7 (75%)	12.8 ± 26.1 (74%)	12.1 ± 27.2 (64%)	18.0 ± 32.2 (78%)

[1] Values are means ± SDs (percentages of consumers) unless otherwise indicated.

Table S10. Intakes of energy, macronutrients, vitamins, and minerals at breakfast among breakfast consumers[1].

	Children (aged 6–11 years)	Adolescents (aged 12–17 years)	Younger adults (aged 18–49 years)	Older adults (aged ≥ 50 years)
n	**1434**	**1104**	**6028**	**13135**
Energy (kcal)	374 ± 157	464 ± 199	411 ± 218	472 ± 194
**Macronutrients (g)**				
Protein	13.1 ± 6.6	15.9 ± 7.8	14.1 ± 8.9	17.5 ± 9.3
Fat	11.8 ± 8.4	13.9 ± 9.1	11.9 ± 9.8	12.1 ± 8.7
SFA	3.9 ± 3.2	4.5 ± 3.4	3.6 ± 3.3	3.5 ± 3.0
MUFA	4.0 ± 3.3	4.7 ± 3.6	4.0 ± 3.9	3.9 ± 3.4
n-6 PUFA	1.9 ± 1.4	2.3 ± 1.7	2.2 ± 2.0	2.4 ± 1.9
n-3 PUFA	0.3 ± 0.3	0.4 ± 0.4	0.4 ± 0.5	0.5 ± 0.6
Carbohydrate	52.7 ± 23.0	67.3 ± 32.2	60.9 ± 32.9	72.5 ± 30.6
Added sugar	5.3 ± 7.8	5.3 ± 7.7	4.9 ± 7.5	5.1 ± 7.1
Dietary fiber	2.2 ± 1.6	2.6 ± 1.8	2.8 ± 2.2	4.4 ± 2.9
**Macronutrient balance (% energy at breakfast)**				
Protein	13.9 ± 3.8	13.8 ± 4.3	13.7 ± 4.7	14.6 ± 4.3
Fat	27.1 ± 13.0	26.4 ± 12.7	24.2 ± 13.6	22.2 ± 11.6
SFA	8.8 ± 5.6	8.5 ± 5.3	7.5 ± 5.7	6.5 ± 4.8
MUFA	9.0 ± 5.4	8.7 ± 5.2	7.9 ± 5.6	7.0 ± 4.8
PUFA	5.1 ± 3.0	5.0 ± 3.0	5.0 ± 3.6	5.3 ± 3.2
Carbohydrate	58.1 ± 14.9	58.7 ± 14.4	61.4 ± 16.1	62.8 ± 13.7
Added sugar	5.8 ± 8.4	5.1 ± 8.3	6.5 ± 13.3	4.8 ± 7.8
**Vitamins**				
Vitamin A (µg RAE)	90.9 ± 100.9	117.2 ± 290.3	96.9 ± 242.2	141.7 ± 344.2
Vitamin D (µg)	0.9 ± 1.8	1.2 ± 2.7	1.2 ± 3.3	2.1 ± 4.5
Vitamin E (mg)	1.1 ± 0.9	1.3 ± 1.1	1.4 ± 1.3	1.7 ± 1.5
Vitamin K (µg)	40.1 ± 71.7	46.5 ± 83.9	55.2 ± 97.7	91.8 ± 121.5
Thiamin (mg)	0.16 ± 0.10	0.20 ± 0.14	0.17 ± 0.14	0.20 ± 0.13
Riboflavin (mg)	0.27 ± 0.18	0.31 ± 0.22	0.27 ± 0.21	0.36 ± 0.23
Niacin (mg)	2.0 ± 2.0	2.6 ± 2.3	2.8 ± 2.6	3.4 ± 2.6
Vitamin B-6 (mg)	0.17 ± 0.14	0.22 ± 0.17	0.21 ± 0.20	0.32 ± 0.20
Vitamin B-12 (µg)	0.9 ± 1.5	1.1 ± 1.7	0.9 ± 2.0	1.5 ± 3.0
Folate (µg)	49.5 ± 35.4	62.5 ± 51.1	65.5 ± 58.0	104.2 ± 74.9
Pantothenic acid (mg)	1.3 ± 0.7	1.5 ± 0.9	1.3 ± 0.9	1.7 ± 1.0
Vitamin C (mg)	15.6 ± 29.5	18.3 ± 24.0	18.4 ± 32.9	34.3 ± 36.5
**Minerals**				
Sodium (g NaCl equivalent)	1.8 ± 1.2	2.2 ± 1.5	2.0 ± 1.6	2.8 ± 1.9
Potassium (mg)	404.0 ± 260.1	486.2 ± 301.1	493.0 ± 342.9	737.8 ± 438.9
Calcium (mg)	127.9 ± 111.6	146.3 ± 122.2	124.0 ± 108.5	176.2 ± 134
Magnesium (mg)	42.5 ± 25.9	52.4 ± 30.6	53.8 ± 35.5	76.8 ± 43.2
Phosphorus (mg)	213.6 ± 120.7	254.2 ± 135.2	221.5 ± 139.9	284.7 ± 155.1
Iron (mg)	1.3 ± 0.9	1.6 ± 1.1	1.6 ± 1.3	2.3 ± 1.6
Zinc (mg)	1.6 ± 0.8	2.0 ± 1.0	1.7 ± 1.1	2.1 ± 1.1
Copper (mg)	0.2 ± 0.1	0.3 ± 0.2	0.3 ± 0.2	0.4 ± 0.2
Manganese (mg)	0.5 ± 0.3	0.7 ± 0.4	0.7 ± 0.6	1.0 ± 0.7

RAE, retinol activity equivalent. [1] Values are means ± SDs unless otherwise indicated.
